# SHH1, a Homeodomain Protein Required for DNA Methylation, As Well As RDR2, RDM4, and Chromatin Remodeling Factors, Associate with RNA Polymerase IV

**DOI:** 10.1371/journal.pgen.1002195

**Published:** 2011-07-21

**Authors:** Julie A. Law, Ajay A. Vashisht, James A. Wohlschlegel, Steven E. Jacobsen

**Affiliations:** 1 Department of Molecular, Cell, and Developmental Biology, University of California Los Angeles, Los Angeles, California, United States of America; 2 Department of Biological Chemistry, David Geffen School of Medicine, University of California Los Angeles, Los Angeles, California, United States of America; 3 Howard Hughes Medical Institute, University of California Los Angeles, Los Angeles, California, United States of America; The University of North Carolina at Chapel Hill, United States of America

## Abstract

DNA methylation is an evolutionarily conserved epigenetic modification that is critical for gene silencing and the maintenance of genome integrity. In *Arabidopsis thaliana*, the *de novo* DNA methyltransferase, DOMAINS REARRANGED METHYLTRANSFERASE 2 (DRM2), is targeted to specific genomic loci by 24 nt small interfering RNAs (siRNAs) through a pathway termed RNA–directed DNA methylation (RdDM). Biogenesis of the targeting siRNAs is thought to be initiated by the activity of the plant-specific RNA polymerase IV (Pol-IV). However, the mechanism through which Pol-IV is targeted to specific genomic loci and whether factors other than the core Pol-IV machinery are required for Pol-IV activity remain unknown. Through the affinity purification of NUCLEAR RNA POLYMERASE D1 (NRPD1), the largest subunit of the Pol-IV polymerase, we found that several previously identified RdDM components co-purify with Pol-IV, namely RNA–DEPENDENT RNA POLYMERASE 2 (RDR2), CLASSY1 (CLSY1), and RNA–DIRECTED DNA METHYLATION 4 (RDM4), suggesting that the upstream siRNA generating portion of the RdDM pathway may be more physically coupled than previously envisioned. A homeodomain protein, SAWADEE HOMEODOMAIN HOMOLOG 1 (SHH1), was also found to co-purify with NRPD1; and we demonstrate that SHH1 is required for *de novo* and maintenance DNA methylation, as well as for the accumulation of siRNAs at specific loci, confirming it is a bonafide component of the RdDM pathway.

## Author Summary

In eukaryotic organisms many systems have evolved to ensure the proper expression of genetic information within each cell, and when these systems malfunction genes can be mis-expressed and cause numerous diseases. One such system involves cytosine DNA methylation, an epigenetic modification that is commonly associated with the repression of transcription and is critical for genome integrity and proper development. In the plant model organism *Arabidopsis thaliana*, DNA methylation is catalyzed by DOMAINS REARRANGED METHYL-TRANSFERASE 2, a homolog of the mammalian *de novo* DNA methyltransferase family, and is targeted to specific loci by small interfering RNAs (siRNAs) through a pathway termed RNA–directed DNA methylation (RdDM). Here we present analysis of our purification of RNA polymerase IV, the polymerase thought to initiate biogenesis of the targeting siRNAs. In addition to the previously identified polymerase IV subunits, we found that several other proteins required for RdDM also co-purify with RNA polymerase IV. Furthermore, we identified a new component required for RdDM, SAWADEE HOMEODOMAIN HOMOLOG 1 (SHH1). Together these findings serve to increase our understanding of DNA methylation by further expanding our knowledge regarding the initial siRNA generating phase of the RdDM pathway.

## Introduction

Epigenetic modifications, including DNA methylation, play important roles in gene regulation and are critical for proper development in most eukaryotic organisms. In *Arabidopsis thaliana*, DNA methylation commonly occurs in all sequence contexts, CG, CHG, and CHH (H = T, A, C). Methylation in the CG context is present in the coding regions of some genes, while methylation in all contexts is present at transposons and other repetitive DNA elements [1]. The *de novo* methyltransferase, DOMAINS REARRANGED METHYLTRANSFERASE 2 (DRM2), is required to establish DNA methylation in all sequence contexts. However, three largely distinct pathways function to maintain DNA methylation in each context [1]: CG methylation is maintained by DNA METHYLTRANSFERASE 1 (MET1), likely during DNA replication in a manner analogous to the mechanism described for CG methylation maintenance in mammals [1]–[3], CHG methylation is maintained by CHROMOMETHYLASE 3 (CMT3) through a reinforcing loop of histone 3 lysine 9 (H3K9) and DNA methylation [1], and CHH methylation is maintained by continual *de novo* methylation by DRM2 in a process termed RNA-directed DNA methylation (RdDM) [1], [4].

Over the last several years many proteins required for RdDM have been identified and characterized, leading to an emerging view of the RdDM pathway [1], [4]. Biogenesis of the targeting siRNAs requires the plant specific Pol-IV polymerase, which is proposed to generate single stranded RNA transcripts [5]. These transcripts are then processed by RNA-DEPENDENT RNA POLYMERASE 2 (RDR2) and DICER-LIKE 3 (DCL3) to generate 24 nt siRNAs that are methylated on their 3′ ends by HUA ENHANCER 1 (HEN1) [6] and loaded into the ARGONAUTE 4 (AGO4), AGO6 and AGO9 effector proteins [1], [4], [7]–[9]. CLASSY 1 (CLSY1), a putative chromatin remodeling factor, is also thought to act in this siRNA generating portion of the pathway [10]. In addition to siRNAs, RdDM is also associated with the presence of intergenic noncoding (IGN) RNA transcripts [11]. The accumulation of IGN transcripts depends on another plant specific RNA polymerase, Pol-V [11], and these transcripts are proposed to act as scaffolds to recruit downstream RdDM effector proteins, which in turn directly or indirectly aid in the recruitment of DRM2 to loci that produce both siRNAs and IGN transcripts. Indeed, both AGO4 and SUPPRESSOR OF TY INSERTION 5-LIKE (SPT5-Like), an AGO4 interacting protein [12], [13], interact with IGN transcripts *in vivo* [12], [14], and INVOLVED IN DE NOVO 2 (IDN2), a protein shown to bind double stranded RNA with a 5′ overhang, is also proposed to act in this downstream portion of the RdDM pathway [15].

Despite these advances in our understanding of the RdDM pathway, the mechanism(s) through which the two plant specific RNA polymerases, Pol-IV and Pol-V, are targeted to specific genomic loci remain largely unknown [5], [16]. Recently, some mechanistic insight into the targeting of Pol-V was provided by the identification a protein complex termed DDR [17] that contains three proteins critical for the production of Pol-V dependent IGN transcripts [11], [14], [17], [18]. This complex is proposed to function at the level of recruitment and/or activation of Pol-V at chromatin and contains three stably associated subunits [17]: DEFECTIVE IN RNA-DIRECTED DNA METHYLATION 1 (DRD1), a putative chromatin remodeling protein [19], DEFECTIVE IN MERISTEM SILENCING 3 (DMS3)/INVOLVED IN DE NOVO 1 (IDN1), a protein with homology to the hinge region of structural maintenance of chromosome (SMC) proteins [15], [20], and RNA-DIRECTED DNA METHYLATION 1 (RDM1) [17], [18]. In addition to the three DDR complex components, two other proteins affect the accumulation of some Pol-V dependent transcripts and siRNAs. The first, termed RNA-DIRECTED DNA METHYLATION 4 (RDM4)/DEFECTIVE IN MERISTEM SILENCING 4 (DMS4), is a protein similar to the yeast protein termed Interacts with Pol II 1 (Iwr1) [21], [22], and the second, termed NUCLEAR RNA POLYMERASE B2 (NRPB2), is a Pol II specific subunit [23]. However, the mechanisms through which these additional factors influence Pol-IV and Pol-V targeting and/or activity awaits further investigation.

To determine whether Pol-IV interacts with any accessory proteins or transcription factors, which may shed light on the mechanism(s) through which it is targeted to transposons and other repetitive DNA elements within the genome, we utilized an epitope tagged version of the largest Pol-IV subunit, NUCLEAR RNA POLYMERASE D1 (NRPD1), to affinity purify the Pol-IV polymerase. In addition to the previously identified Pol-IV subunits [24], we identified several known RdDM components, including RDR2, CLSY1, and RDM4 in our NRPD1 purification, as well as a new RdDM component, SAWADEE HOMEODOMAIN HOMOLOG 1 (SHH1). SHH1 contains a cryptic homeodomain and a SAWADEE domain of unknown function and is required for the accumulation of siRNAs at some loci as well as for both *de novo* and maintenance DNA methylation.

## Results/Discussion

**Figure 1:**
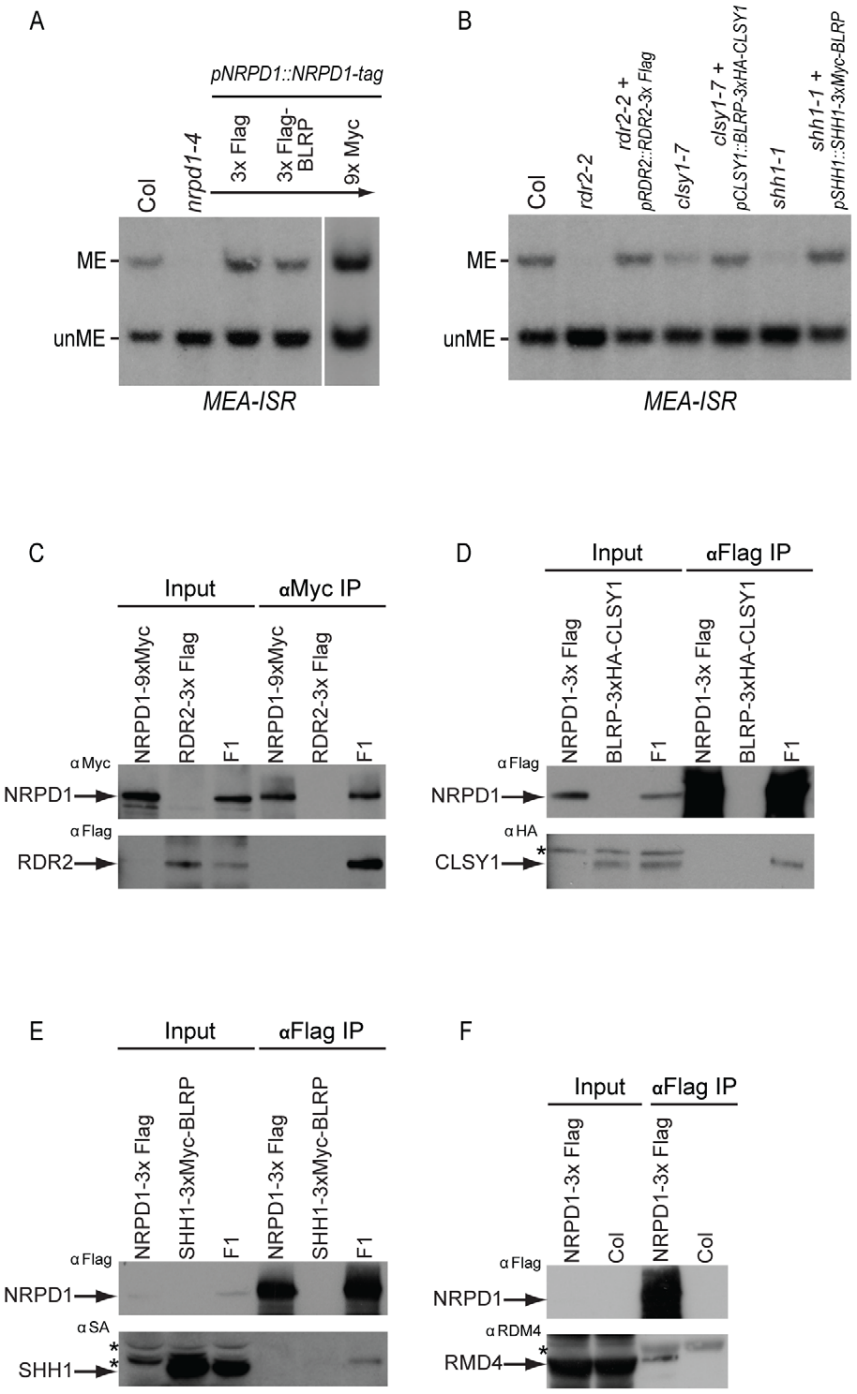
Complementation of DNA methylation defects with epitope tagged RdDM components and co-immunoprecipitation analyses. (A and B) Southern blot analysis of DNA methylation at the *MEA-ISR* locus using genomic DNA digested with the Msp I methylation sensitive restriction enzyme. Bands representing the presence or absence of DNA methylation are indicated as ME or unME, respectively. In (A), genomic DNA was isolated from either wild-type plants of the Columbia (Col) ecotype, homozygous *nrpd1-4* mutant plants, or T_4_ plants homozygous for both the *nrpd1*-4 allele and a transgene containing the indicated carboxy-terminal epitope tagged versions of the *NRPD1* gene under the control of its endogenous promoter (*pNRPD1*). BLRP; Biotin Ligase Recognition Peptide. In (B), genomic DNA was isolated from either Col plants, *shh1-1*, *rdr2-2*, or *clsy1-7* single mutant plants, or T_2_ transgenic plants expressing the indicated epitope tagged versions of SHH1, RDR2, or CLSY1 in their respective homozygous mutant backgrounds. (C–F) Co-immunoprecipitation analyses confirming the interactions between (C) NRPD1 and RDR2, (D) NRPD1 and CLSY1, (E) NRPD1 and SHH1, and (F) NRPD1 and RDM4. For each experiment, lanes containing protein extracted from each of the parental lines and the resultant F_1_ line are indicated below the “input” and “immunoprecipitation (IP)” headings and the antibody (α) used for each western blot is indicated next to each panel (upper left corner). The position of each epitope tagged or endogenous protein is indicated with a closed arrowhead and background bands are indicated by an asterisk (*).

To aid in the purification of Pol-IV, constructs encoding epitope tagged versions of the largest Pol-IV subunit, NRPD1, were generated (Table S1) and transformed into the null *nrpd1-4* mutant background. Transgenic plant lines containing either an *NRPD1-3×Flag* or an *NRPD1-3×Flag-Biotin Ligase Recognition Peptide (BLRP)* transgene were able to complement the DNA methylation defects observed at the *MEA-ISR* locus in the *nrpd1-4* mutant, restoring DNA methylation to a level similar to that observed in the wild-type Columbia (Col) ecotype (Figure 1A). These complementing transgenic lines were then used to affinity purify NRPD1 from floral tissue and mass spectrometric (MS) analyses were used to reveal the identity of co-purifying proteins (Table 1 and Figure S1). As expected, the MS analyses revealed peptides corresponding to many known Pol-IV subunits [24]. However peptides corresponding to several other RNA-directed DNA methylation components, as well as a previously uncharacterized protein, At1g15215, were also identified (Table 1).

**Table 1:** Mass-spectrometric analysis of NRPD1 affinity purifications.

Pol IV subunits							
Protein	Re-designation	AGI code	Spectra	Unique Peptides	% Coverage	NSAF	% NRPD1
NRPD1		At1g63020	363	72	38	1178.1	100
NRPD2/E2		At3g23780	274	51	24.5	1130.6	96
NRPB3/D3/E3A		At2g15430	125	23	44.2	1847.8	157
NRPE3B	NRPD3B/E3B	At2g15400	46	13	24.8	680.0	58
NRPD4/E4		At4g15950	23	3	12.7	677.9	58
NRPB5/D5		At3g22320	60	14	44.9	1380.2	117
NRPE5B	NRPD5B/E5B	At2g41340	17	8	35.8	367.7	31
NRPB6A/D6A/E6A		At5g51940	4	2	17.4	131.0	11
NRPD7	NRPD7A	At3g22900	10	3	19.5	271.0	23
NRPE7	NRPD7B/E7B	At4g14660	2	2	14.6	53.0	4
NRPB8B/D8B/E8B		At3g59600	0	0	0	0.0	0
NRPB9A/E9A	NRPB9A/D9A/E9A	At3g16980	28	8	43.9	1158.2	98
NRPB9B/D9B/E9B		At4g16265	37	7	46.5	1530.5	130
NRPB10/D10/E10		At1g11475	29	2	12.7	1926.1	163
NRPB11/D11/E11		At3g52090	21	6	26	853.7	72
NRPB12/D12/E12		At5g41010	10	7	74.5	924.6	78
**Other RdDM components**							
RDR2		At4g11130	195	38	29.7	811.6	69
DMS4/RDM4		At2g30280	20	7	11.6	272.6	23
CLSY1		At3g42670	27	14	8	101.4	9
CLSY2		At5g20420	28	14	11.8	104.7	9
CHR31	CLSY3	At1g05490	40	20	16.9	133.8	11
CHR40	CLSY4	At3g24340	25	15	13.8	104.1	9
SHH1		At1g15215	13	6	25.2	237.6	20

Table summarizing the polymerase subunits (upper) and other known or putative RNA directed DNA methylation (RdDM) components (lower) found to co-purify with NRPD1. Based on these MS analyses, several proteins have been given the additional designation of NRP “D#”, to indicate they are also present in the Pol-IV polymerase. The percent coverage (% coverage) column takes into account peptides that map only to the AGI code indicated in the third column (see also Figure S1). The percentage of NRPD1 (% NRPD1) column indicates the approximate stoichiometry of each co-purifying protein as a function of the Normalized Spectral Abundance Factor (NSAF)×10e5 [43].

While the overall subunit composition of the Pol-IV polymerase identified in this study is in strong agreement with that of the previous purification [24] there were a few notable differences. In two independent experiments our purification failed to yield peptides corresponding to the NRPB8B/D8B/E8B subunit, although peptides corresponding to all the other Pol-IV subunits identified by Ream et al. [24] were identified (Table 1). In addition, while Ream et al. [24] reported that the percent coverage of the NRPE7 subunit in their Pol-IV purification was much lower than observed for the NRPD7 subunit, and concluded that NRPD7 is the predominant subunit associated with Pol-IV, we found that the percent coverage of these two proteins was roughly similar (Table 1 and Figure S1). Similarly, we found that the NRPE3B and NRPB3/D3/E3A subunits were both covered to similar extents. Finally, although no peptides corresponding to the NRPB9A/E9A subunit were recovered by Ream et al. [24], we found that the NRPB9A/E9A and NRPB9B/D9B/E9B subunits were both covered to similar extents. We also identified peptides corresponding to NRPE5B (At2g41340) (Table 1), one of the three NRPE5-like proteins (also designated NRPE5A, NRPE5B, and NRPE5C) present in the *Arabidopsis* RPB5 family of RNA polymerase subunits [25]. Taken together our affinity purification data demonstrates that the NRPE3B, NRPE5B, NRPE7, and NRPB9A/E9A subunits can all associate with the Pol-IV polymerase and should be re-designated NRPD3B/E3B, NRPD5B/E5B, NRPD7B/E7B, and NRPB9A/D9A/E9A (Table 1). The identification of these polymerase subunits in our Pol-IV purification could reflect the less stringent ionic conditions used for our purification and may suggest these proteins are less stably associated with the core Pol-IV machinery than some of the other subunits.

In addition to Pol-IV subunits, peptides corresponding to several known RdDM components were also identified as co-purifying with epitope tagged NRPD1. These components include RDR2, an RNA-dependent RNA polymerase [26], RDM4, an IWR-type transcription factor [21], [22], and CLSY1, a putative chromatin remodeling factor [10] (Table 1). Three other putative chromatin remodeling factors, specifically CLSY2 (At5g20420), CHROMATIN REMODELING 31 (CHR31) (At1g05490), and CHR40 (At3g24340), which are all closely related to CLSY1 [10], were also identified (Table 1), suggesting they may also interact with Pol-IV and may play a role in DNA methylation. Based on their co-purification with Pol-IV, and their phylogenetic relationship with CLSY1 and CLSY2, we herein designate CHR31 and CHR40 as CLASSY3 (CLSY3) and CLASSY4 (CLSY4), respectively.

In order to confirm the interactions between the known RdDM components and NRPD1, co-immunoprecipitation (co-IP) experiments were conducted utilizing either an RDM4 antibody or F_1_ transgenic plant lines expressing complementing epitope tagged versions of NRPD1 and either RDR2 or CLSY1 (Figure 1B). Consistent with the MS analyses, these experiments demonstrate that RDR2, CLSY1, and RDM4 co-purify with NRPD1 (Figure 1C, 1D, 1F). Notably, while these RdDM components are all thought to act in the upstream, siRNA generating portion of the RdDM pathway, their co-purification with the NRPD1 subunit of Pol-IV provides the first evidence that they may physically associate with each other and/or with the Pol-IV machinery. However, further biochemistry will be required to determine whether all these upstream RdDM components are present in the same complex with Pol-IV or whether multiple Pol-IV complexes are present and possibly functioning in a locus specific manner.

Affinity purification of epitope tagged NRPD1 also yielded peptides that correspond to an uncharacterized protein, At1g15215 (Table 1), suggesting that this protein may function in the RdDM pathway. *At1g15215* encodes a protein of ∼30 KDa which contains a cryptic homeodomain and a SAWADEE domain, placing it in the SAWADEE class of plant specific homeobox transcription factor genes [27]. Although the function of the SAWADEE domain is unknown, the presence of conserved cysteine and histidine residues suggests it may function in binding DNA [27]. The *Arabidopsis* genome encodes one other protein within this class of homeodomain proteins, At3g18380, but no peptides specific to this protein were identified in the MS analysis. At1g15215 and At3g18380 have the same domain architecture, are 53% similar and 41% identical and have homologs in moss, *Selaginella*, and other flowering plants [27] and are herein named SAWADEE HOMEODOMAIN HOMOLOG 1 (SHH1) and SAWADEE HOMEODOMAIN HOMOLOG 2 (SHH2), respectively. In order to confirm the interaction between SHH1 and NRPD1, a co-IP experiment was conducted (Figure 1E) using F_1_ plants expressing complementing, epitope tagged versions of both NRPD1 and SHH1 (Figure 1A, 1B). Consistent with the MS analyses, this experiment demonstrated that SHH1 and NRPD1 co-immunoprecipitate (Figure 1E).

### The homeodomain protein, SHH1, is required for maintenance and *de novo* DNA methylation

**Figure 2:**
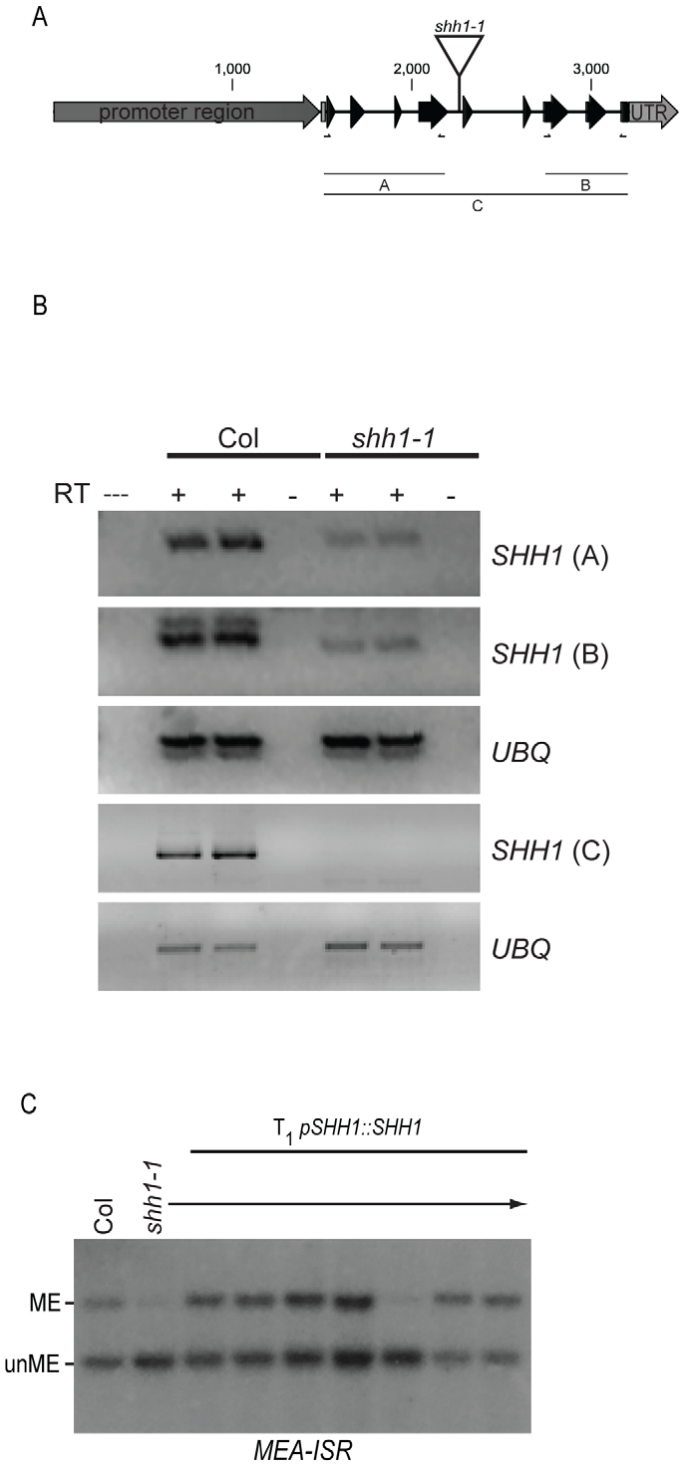
Characterization of the *shh1-1* allele and genomic complementation. (A) Cartoon representation of the *SHH1* locus. The promoter and UTRs are shown in grey and the exons are shown as black arrows. The location of the T-DNA insertion in the *shh1-1* mutant is indicated and the three regions, A, B, and C, amplified by Reverse-Transcriptase PCR are indicated below. (B) Analysis of *SHH1* expression in the *shh1-1* allele by Reverse Transcriptase followed by gene specific PCR. Three primer sets, (A), (B), and (C), within the *SHH1* gene were assessed (see Table S2). As a loading control, primers amplifying a ubiquitin gene (*UBQ*) were also used (see Table S2). Reactions lacking (-) the Reverse Transcriptase (RT) were included to ensure that no DNA contamination was present and reactions containing (+) the RT were conducted in duplicate. (---) indicates the PCR reaction was conducted in the absence of cDNA. (**C**) *MEA-ISR* Southern blot as described in Figure 1, showing complementation of the *shh1-1* mutant phenotype with a genomic *SHH1* transgene (*pSHH1::SHH1*) using genomic DNA extracted from wild-type Col plants, *shh1* mutants, or individual T_1_ transformants in the *shh1-1* mutant background.

**Figure 3:**
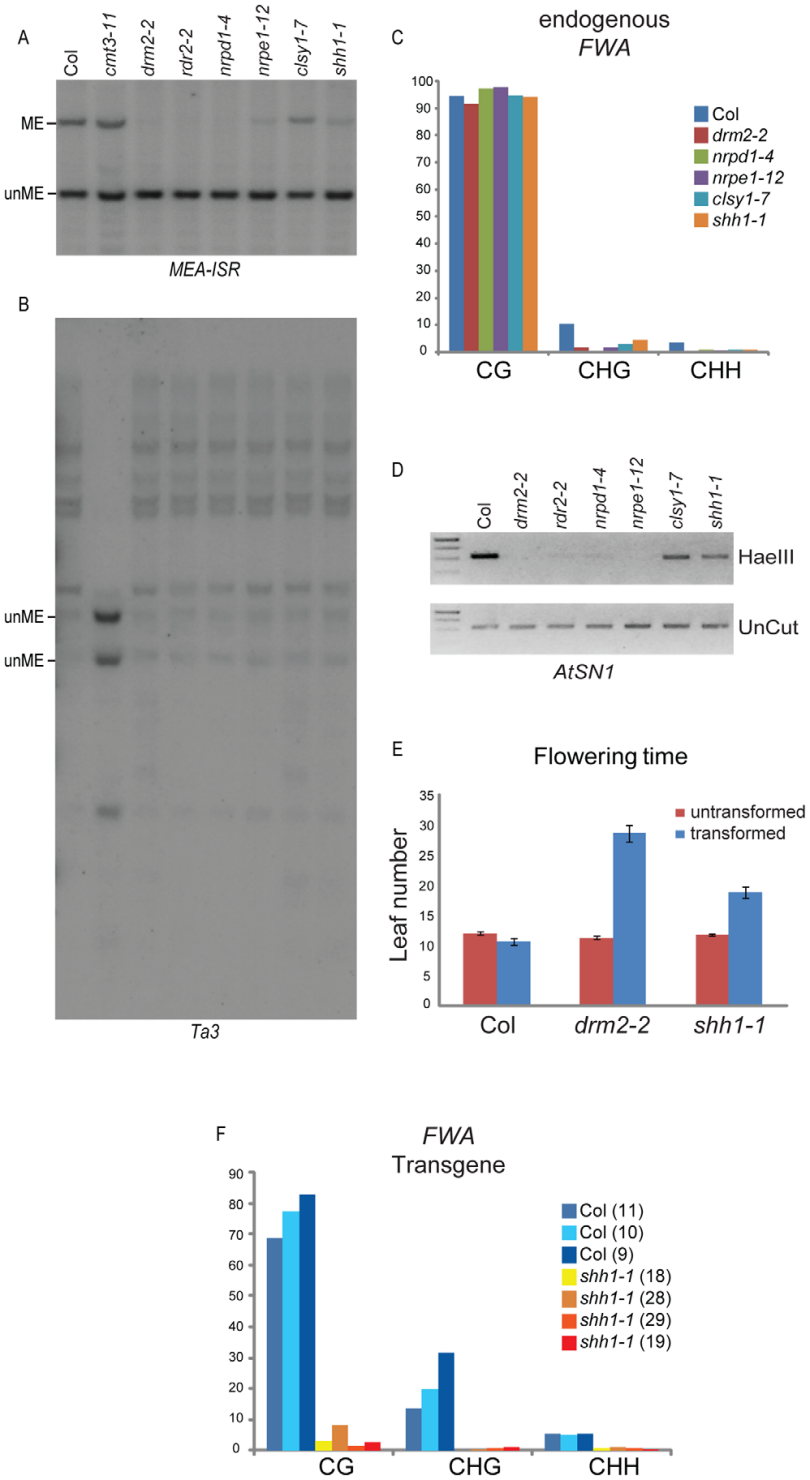
Characterization of methylation defects in the *shh1* mutant. Assessment of DNA methylation levels at the (A) *MEA-ISR* and (B) *Ta3* loci by Southern blotting using Msp I digested genomic DNA extracted from the genotypes indicated above each lane and a locus-specific radiolabeled probe. ME; methylated DNA, unME; unmethylated DNA. (C) Assessment of the levels of DNA methylation at the endogenous *FWA* locus by bisulfite sequencing. The y axis is the percent of methylation and the x-axis is the context of methylation. (D) Assessment of the level of DNA methylation at the *AtSN1* locus using a methyl-cutting assay in which genomic DNA extracted from the indicated genotypes was either untreated (unCut) or digested with the methylation sensitive Hae III enzyme prior to amplification of the *AtSN1* locus. (E and F) Assessment of the role of *SHH1* in *de novo* DNA methylation by flowering time (E) and bisulfite sequencing (F). In (E), the graph shows the leaf number (y axis) of individual T_1_ plants of the indicated genotype (x axis) either untransformed or transformed with a transgene carrying the *FWA* gene. The black bars represent standard error. In (F), T_2_ tissue from individual Col or *shh1* T_1_ transformants was used to assess the level of DNA methylation on the *FWA* transgene. The y axis is the percent of methylation observed and the x-axis is the context of methylation. The number in parentheses next to the plant genotype indicates the flowering time of the T_1_ parent.

To determine whether SHH1 is required for DNA methylation, a T-DNA insertion mutant, *shh1-1* (Salk_074540C), was obtained and the *SHH1* transcript levels in this mutant were assessed by semi-quantitative Reverse Transcriptase PCR assays (Figure 2). In the mutant line, the abundance of transcripts corresponding to the 5′ and 3′ portions of the *SHH1* gene were reduced and the full-length transcript was undetectable, suggesting that no wild-type SHH1 protein is produced in this mutant (Figure 2B). This allele was then used to assess the levels of DNA methylation relative to several known DNA methylation mutants at loci controlled by each of the three *Arabidopsis* DNA methyltransferases (DRM2, CMT3, and MET1) by Southern blotting, bisulfite sequencing and methyl-sensitive PCR cutting assays. At a DRM2 controlled locus, *MEA-ISR*, mutation of *SHH1* causes a strong decrease in DNA methylation, reducing the level of methylation to near the level observed in the *drm2* mutant (Figure 3A). At a CMT3 controlled locus, *Ta3*, mutation of *SHH1* had no effect on DNA methylation (Figure 3B). However, at a locus controlled by both DRM2 and CMT3, *AtSN1*, mutation of *SHH1* again resulted in reduced DNA methylation (Figure 3D). At the *FWA* locus, which is controlled by MET1, the level of CG methylation was not reduced in the *shh1* mutant, however, like observed in *drm2* and other RdDM mutants, the levels of non-CG methylation were reduced (Figure 3C). To confirm that the decreases in DNA methylation observed in the *shh1* mutant were indeed due to disruption of the *SHH1* locus, *shh1* plants were transformed with a construct containing the *SHH1* gene, including its upstream promoter region, and DNA methylation was assessed at the *MEA-ISR* locus. In the majority of the resulting T_1_ plant lines DNA methylation was restored to the wild-type level (Figure 2C). Together, these DNA methylation analyses demonstrate that SHH1 is required for DNA methylation and, consistent with its co-purification with NRPD1, it appears to be specific for the DRM2-mediated DNA methylation pathway.

In *Arabidopsis*, the DRM2 pathway is also required for *de novo* DNA methylation. To assess *de novo* DNA methylation, an *FWA* transgene transformation assay is often employed [28], [29]. In this assay, an *FWA* transgene, the expression of which is controlled by DNA methylation, is stably introduced into the *Arabidopsis* genome. If *de novo* methylation occurs the transgene is silenced, but if the *de novo* methylation pathway is impaired the transgene is expressed, leading to a delay in flowering that can be scored as an increase in the number of rosette leaves produced prior to flowering. Upon introduction of an *FWA* transgene, *shh1* mutant plants flowered an average of seven leaves later than untransformed plants (Figure 3E), demonstrating that *SHH1* is required to silence the incoming transgene. Furthermore, bisulfite sequencing of the *FWA* transgene from several wild type Col and *shh1* transformants in the T_2_ generation confirmed that *de novo* methylation was impaired in the *shh1* mutant (Figure 3F). However, consistent with the partial phenotype observed for maintenance methylation (Figure 3A, 3C, 3D), flowering was not as delayed in the *shh1* mutant as was observed for the *drm2* mutant (Figure 3E).

### SHH1 is required for the accumulation of siRNAs but not for the production of Pol-V–dependent transcripts

**Figure 4:**
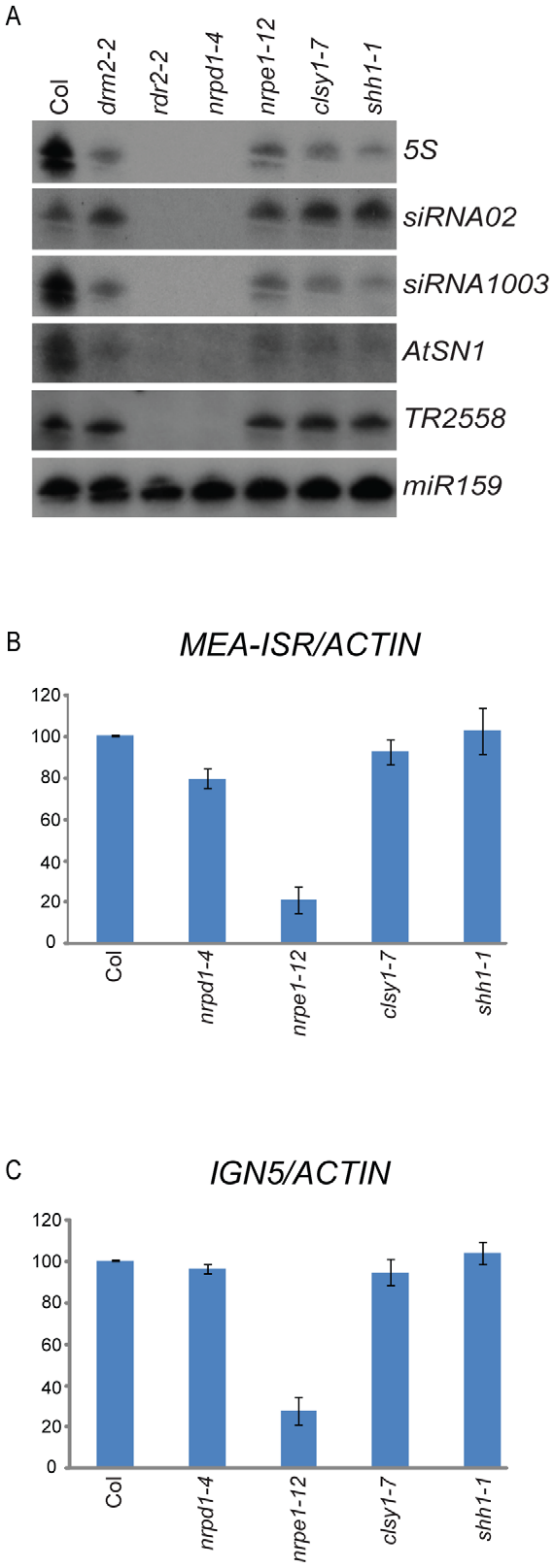
Characterization of siRNAs and Pol-V dependent transcripts in the *shh1* mutant. (A) Assessment of siRNA levels at the indicated loci (right) by northern blotting using RNA extracted from the genotypes indicated above. (B–C) Assessment of Pol-V dependent transcript levels (y axis) using RNA isolated from the genotypes indicated (x axis) relative to *ACTIN* and normalized to Col at the *MEA-ISR* and *IGN5* loci. Thick blue bars represent the average of three biological replicates and thin black bars are the standard error of the three experiments.

To gain insight into where in the RdDM pathway SHH1 functions, the production of siRNAs and Pol-V dependent noncoding RNA transcripts were assessed in the *shh1* mutant. Consistent with the co-purification of SHH1 with the NRPD1 subunit of Pol-IV, siRNAs levels at some loci were significantly reduced in the *shh1* mutant (Figure 4A), as was previously observed for *clsy1* [10], another weak DNA methylation mutant [30]. These findings demonstrate that SHH1 plays an important role in the accumulation of siRNAs.

To determine whether SHH1 also functions in the accumulation of Pol-V dependent IGN transcripts, the levels of such transcripts at the *MEA-ISR* and *IGN5* loci were assessed. In the *clsy1* and *shh1* mutants the levels of Pol-V dependent transcripts were unaffected in three biological replicates (Figure 4B, 4C), suggesting that these RdDM components may be specific to the siRNA generating portion of the RdDM pathway.

### Conclusion

Through the affinity purification of the NRPD1 subunit of the Pol-IV polymerase we were able to further refine the subunit composition of Pol-IV and identify two putative additional components, NRPB9A/D9A/E9A and NRPD5B/E5B. In addition to Pol-IV subunits, our purification also yielded peptides corresponding to several known RdDM components, which like Pol-IV are thought to function in the upstream, siRNA generating portion of the RdDM pathway. These proteins include the RDM4 transcription factor, the CLSY1 putative chromatin remodeling protein, and RDR2, the RNA-dependent RNA polymerase shown previously to be required for siRNA biogenesis. The finding that these upstream RdDM components co-purify with Pol-IV suggests that this portion of the pathway may be more physically coupled than previously envisioned. Indeed, the approximate stoichiometry of NRPD1 and RDR2 is near 1∶1 (Table 1) and it is likely that tight coupling of the activities of Pol-IV and RDR2 is biologically relevant. For example, this could restrict the activity of RDR2 to transcripts being produced specifically by Pol-IV, thereby reducing the chances that transcripts from other polymerases would be copied into double stranded RNA and channeled into the siRNA-directed DNA methylation pathway, which could lead to off-target DNA methylation and gene silencing.

Our affinity purification of NRPD1 also led to the identification of a new component of the RdDM pathway, SHH1. Mutations in *SHH1* result in decreased DNA methylation at loci controlled by the RdDM pathway and in reduced levels of siRNAs, suggesting that SHH1 may function early in the RdDM pathway. Although it is tempting to speculate that SHH1 may be involved in the targeting and/or recruitment of the Pol-IV polymerase to chromatin, as it possesses both a homeodomain and a SAWADEE domain, further experiments will be required to determine whether SHH1 interacts with chromatin and whether it plays a role in the recruitment of Pol-IV to silenced loci.

## Materials and Methods

### Plant materials

Plants were grown under long day conditions and the following previously characterized T-DNA insertion mutant lines in the Col ecotype were utilized: *cmt3-11* (SALK_148381) [31], *drm2-2* (SALK_150863) [31], *rdr2-2* (SALK_059661) [32], *nrpd1-4* (SALK_083051) [33], *nrpe1-12* (SALK_033852) [34], *clsy1-7* (SALK_018319) [35]. Characterization of the Col *shh1-1* (SALK_074540C) T-DNA insertion allele is described in Figure 2.

### Generation of gateway entry clones, destination clones, and transgenic *Arabidopsis* plant lines

DNA fragments containing the *NRPD1*, *RDR2*, *CLSY1*, or *SHH1* genes, including their endogenous promoter regions, were amplified by PCR using the primers listed in Table S2. For *NRPD1*, a pEarlyGate302 plasmid [36] containing the NRPD1 gene and promoter [37] was used as the DNA template while for *RDR2*, *CLSY1*, and *SHH1* genomic DNA isolated from the Col ecotype served as the DNA template. PCR products were cloned into the pENTR/D-TOPO vector (Invitrogen) per manufacturer instructions. For *NRPD1*, *SHH1* and *RDR2*, carboxy-terminal tags (Table S1) were inserted into a 3′ Asc I site present in the pENTR/D-TOPO vector. For *CLSY1*, an amino-terminal BLRP-3×HA tag (Table S1) was inserted into a Kpn I restriction site engineered into the *CLSY1* genomic sequence upstream of the start codon through quickchange site directed mutagenesis (Stratagene) per manufacturer instructions.

The described pENTR/D constructs were digested with the Mlu I restriction enzyme and then recombined into one of two modified gateway destination vectors, which differ only in their plant drug resistance gene, using LR Clonase (Invitrogen) per manufacturer instructions. For the *NRPD1* constructs the destination vector used contains a gene conferring resistance to the BASTA herbicide and for the *RDR2*, *SHH1* and *CLSY1* constructs the destination vector used contains a gene conferring resistance to Hygromycin. Both destination vectors are based on the pEarleyGate302 vectors described in [36] but were modified as previously described [38], [39], such that they contain the BirA gene, the product of which transfers a biotin group onto a lysine residue present in the BLRP epitope tag, under the control of an *ACTIN* promoter. These destination vectors were then transformed into the AGLO strain of *Agrobacterium* by electroporation. *Arabidopsis* plants carrying the *nrpd1-4, shh1-1, rdr2-2, or clsy1-7* mutant alleles were transformed with the various *NRPD1*, *SHH1*, *RDR2*, or *CLSY1* epitope tagged constructs, respectively, by the floral dip method as described in [40]. Transformed plants were selected using either BASTA or hygromycin and transformants containing only a single insertion site were determined by segregation analysis in the subsequent generation.

### Affinity purification and mass-spectrometric analyses

Approximately 10 g of flower tissue from NRPD1-3×Flag and NRPD1-3×Flag-BLRP transgenic T_4_ plants, or from Col plants as a negative control, were ground in liquid nitrogen, and resuspended in 50 mL of lysis buffer (LB: 50 mM Tris pH7.6, 150 mM NaCL, 5 mM MgCl_2_, 10% glycerol, 0.1% NP-40, 0.5 mM DTT, 1 µg/µL pepstatin, 1 mM PMSF and 1 protease inhibitor cocktail tablet (Roche, 14696200)). The tissue was then homogenized by douncing and centrifuged at 4°C in an SS34 rotor for 25 minutes at 12,500 rpm. Each supernatant was incubated at 4°C for 2.5 hours with 200 µL of Dynabeads (M-270 Epoxy, Invitrogen, 143.01) conjugated with a Flag antibody (Sigma F 3165) according to manufacturer instructions. The Flag beads were then washed twice with 40 mL of LB and five times with 1 mL of LB. For each wash, the beads were rotated at 4°C for 5 minutes. Proteins were then released from the Flag beads during five room temperature incubations with 150 µL of 3×Flag peptide (Sigma, F 4799) at a concentration of 100 µg/mL.

Mass spectrometric analyses were conducted as described in [17]. For comparison with the previously published Pol-IV affinity purification and MS analyses [24], peptide coverage maps (Figure S1) were generated and the percent coverage (Table 1: “% Coverage”) of each Pol-IV subunit was calculated using only the uniquely mapping peptides recovered from the MS analysis, as was done in Ream et al. [24].

### Co-immunoprecipitation analyses

For the co-IP experiments between NRPD1 and RDR2, CLSY1, and SHH1, 0.5 g–1 g of tissue from each parental line as well as F_1_ plants expressing complementing, epitope tagged versions of both proteins were used. For the co-IP between NRPD1 and RDM4, 1 g of tissue from either Col plants or plants expressing a complementing, epitope tagged version of NRPD1 was used. For each experiment, the tissue as ground in liquid nitrogen with lysis buffer (LB) (2.5 mL per 0.5 g of tissue) and the lysate was cleared by centrifugation at 13,200 rpm in microfuge tubes for 10 minutes at 4°C. The supernatants were incubated with 100 µL of either Myc agarose (50% slurry Covance AFC-150P) or M2 Flag agarose (50% slurry, Sigma A2220) beads for 2 hours at 4°C with rotation. The beads were then washed 5 times, for 5 minutes, with 1 mL of LB and resuspended in 50 µL of SDS-PAGE loading buffer. 35 µL or 9 µL of input and bead eluate were run on 4–12% SDS-PAGE gels in Figure 1D–1F or Figure 1C, respectively, and the various proteins were detected by western blotting using either ANTI-FLAG M2 Monoclonal Antibody-Peroxidase Conjugate (Sigma A 8592) at a dilution of 1∶5000, c-Myc 9E10 mouse monoclonal antibody (Santa Cruz Biotechnology, sc-40) at a dilution of 1∶5000, or anti-RDM4 at a dilution of 1∶2500. Goat anti-mouse IgG horseradish peroxidase (Thermo scientific, 31430) or goat anti-rabbit IgG horseradish peroxidase (Thermo scientific, 31460) was used at a dilution of 1∶5000 as the secondary antibody. All westerns were developed using ECL Plus Western Blotting Detection System (GE healthcare RPN2132).

### DNA methylation

Genomic DNA isolation and Southern blot analyses at the *MEA-ISR* and *Ta3* loci were conducted as described in [38]. Bisulfite treatment of genomic DNA, amplification of the *FWA* endogene, cloning and sequencing of the resulting PCR products were as described in [38]. For the Col control and each mutant ∼10–15 clones were analyzed. For bisulfite analysis of the *FWA* transgene, genomic DNA was extracted from pooled T_2_ plants from individual T_1_ transformants and digested with the Bgl II restriction enzyme, which specifically cuts within the *FWA* endogene, prior to bisulfite conversion. The *AtSN1* cutting assay was conducted as described in [41] except the uncut samples were amplified for 25 cycles, the cut samples for 32 cycles, and the amplification products were visualized by agarose gel electrophoresis. *FWA* transformation, T_1_ selection and flowering time analysis were as describe in [15].

### RNA analysis

siRNAs for northern blotting were isolated as follows: 1 g of flower tissue from each genotype was ground in liquid nitrogen with a mortar and pestle, incubated with 10 mL of trizol reagent (Invitrogen 15596-026) at room temperature for 10 minutes, and mixed with 2 mL of chloroform. The samples were then centrifuged at 13,000 rpm for 30 minutes at 4°C and the supernatants were mixed with one volume of cold isopropanol. Samples were then centrifuged at 13,000 rpm for 30 minutes at 4°C and the pelleted RNA was resuspended in 500 µL of DEPC-treated water. These total RNA preparations were then enriched for small RNAs through a polyethelene glycol (PEG) precipitation step. One volume of a 20% PEG 8000/2M NaCl solution was added to each RNA preparation and then centrifuged at 13,000 rpm for 15 minutes at 4°C. The supernatant, containing the small RNA molecules, was collected and precipitated with 0.8 volumes of cold isopopanol as described above. ∼30 µg of the resulting small RNA samples were run on 15% polyacrlyamide-7M Urea gels and transferred to Hybond-NX membranes (Amersham RPN303T). Membranes were blocked using 10 mL of ULTRhyb-Oligo buffer (Ambion AM8663) and probed with 5′ end radiolabeled oligos as described in [42].

*SHH1* expression was assessed by Reverse Transcriptase PCR using total RNA extracted from 100 mg of flower tissue using the Trizol reagent and cDNAs were synthesized using Super ScriptII (Invitrogen) per manufacturer instructions.

Detection of Pol-V dependent transcripts at the *MEA-ISR* and *IGN5* loci were conducted as described in [17]. The data represents three biological replicates with standard errors. To quantify the levels of each transcript the signal from the *ACTIN*, *MEA-ISR*, and *IGN5* primer pairs were determined relative to a standard curve generated using sonicated DNA from Col plants. The levels of the *MEA-ISR* and *IGN5* transcripts where then normalized to the level of the *ACTIN* transcript. Since a different standard curve was used for each of the three different biological replicates, the values for *MEA-ISR/ACTIN* and *IGN5/ACTIN* for each genotype within a single biological replicate were normalized to the signal of *MEA-ISR/ACTIN* and *IGN5/ACTIN* observed for the Col sample from the same biological replicate (with this signal being set to 100), allowing comparison of the three different sets of data.

## Supporting Information

Figure S1 Peptide coverage maps of each Pol IV subunit and other co-purifying RdDM components. Listed below are the gene names and AGI numbers for each protein listed in Table 1 followed by their full length protein sequence. The peptides recovered from the MS analysis are listed and mapped onto the protein sequence. Regions that are crossed out correspond to peptides that also maps to another gene while regions that are highlighted in yellow correspond to a unique peptide (i.e. a peptide that maps only to the listed protein sequence) and regions in green correspond to two unique peptides.
(DOC)

Table S1 Amino acid sequences of epitope tags. The amino acid sequence of each tandem affinity epitope tag are shown with the Biotin Ligase Recognition Peptide (BLRP) in bold type, the L to R mutation in the mutated BLRP tag in red, the 3C protease cleavage site underlined and the Flag, Myc or HA tag in large, non-bold text. * indicates a stop codon.
(DOC)

Table S2 DNA sequences of primers and quantitative PCR probes.
(DOC)
