# Supplementary material for: SHH1, a Homeodomain Protein Required for DNA Methylation, As Well As RDR2, RDM4, and Chromatin Remodeling Factors, Associate with RNA Polymerase IV
Source: PLoS Genet. 2011 Jul 21;7(7):e1002195. doi: 10.1371/journal.pgen.1002195 (PMC3141008; doi:10.1371/journal.pgen.1002195)
Supplement: Figure S1 — Peptide coverage maps of each Pol IV subunit and other co-purifying RdDM components. Listed below are the gene names and AGI numbers for each protein listed in Table 1 followed by their full length protein sequence. The peptides recovered from the MS analysis are listed and mapped onto the protein sequence. Regions that are crossed out correspond to peptides that also maps to another gene while regions that are highlighted in yellow correspond to a unique peptide (i.e. a peptide that maps only to the listed protein sequence) and regions in green correspond to two unique peptides. (DOC) [file pgen.1002195.s001.doc]

Figure S1 **Peptide coverage maps of each Pol IV subunit and other co-purifying components.** Listed below are the gene names and AGI numbers for each protein listed in Table 1 followed by their full length protein sequence. The peptides recovered from the MS analysis are listed and mapped onto the protein sequence. Regions that are crossed out correspond to peptides that also maps to another gene while regions that are highlighted in yellow correspond to a unique peptide ( i.e. a peptide that maps only to the listed protein sequence) and regions in green correspond to two unique peptides.

**NRPD1 (At1g63020)**

MEDDCEELQVPVGTLTSIGFSISNNNDRDKMSVLEVEAPNQVTDSRLGLPNPDSVCRTCGSKDRKVCEGHFGVINFAYSIINPYFLKEVAALLNKICPGCKYIRKKQFQITEDQPERCRYCTLNTGYPLMKFRVTTKEVFRRSGIVVEVNEESLMKLKKRGVLTLPPDYWSFLPQDSNIDESCLKPTRRIITHAQVYALLLGIDQRLIKKDIPMFNSLGLTSFPVTPNGYRVTEIVHQFNGARLIFDERTRIYKKLVGFEGNTLELSSRVMECMQYSRLFSETVSSSKDSANPYQKKSDTPKLCGLRFMKDVLLGKRSDHTFRTVVVGDPSLKLNEIGIPESIAKRLQVSEHLNQCNKERLVTSFVPTLLDNKEMHVRRGDRLVAIQVNDLQTGDKIFRSLMDGDTVLMNRPPSIHQHSLIAMTVRILPTTSVVSLNPICCLPFRGDFDGDCLHGYVPQSIQAKVELDELVALDKQLINRQNGRNLLSLGQDSLTAAYLVNVEKNCYLNRAQMQQLQMYCPFQLPPPAIIKASPSSTEPQWTGMQLFGMLFPPGFDYTYPLNNVVVSNGELLSFSEGSAWLRDGEGNFIERLLKHDKGKVLDIIYSAQEMLSQWLLMRGLSVSLADLYLSSDLQSRKNLTEEISYGLREAEQVCNKQQLMVESWRDFLAVNGEDKEEDSVSDLARFCYERQKSATLSELAVSAFKDAYRDVQALAYRYGDQSNSFLIMSKAGSKGNIGKLVQHSMCIGLQNSAVSLSFGFPRELTCAAWNDPNSPLRGAKGKDSTTTESYVPYGVIENSFLTGLNPLESFVHSVTSRDSSFSGNADLPGTLSRRLMFFMRDIYAAYDGTVRNSFGNQLVQFTYETDGPVEDITGEALGSLSACALSEAAYSALDQPISLLETSPLLNLKNVLECGSKKGQREQTMSLYLSEYLSKKKHGFEYGSLEIKNHLEKLSFSEIVSTSMIIFSPSSNTKVPLSPWVCHFHISEKVLKRKQLSAESVVSSLNEQYKSRNRELKLDIVDLDIQNTNHCSSDDQAMKDDNVCITVTVVEASKHSVLELDAIRLVLIPFLLDSPVKGDQGIKKVNILWTDRPKAPKRNGNHLAGELYLKVTMYGDRGKRNCWTALLETCLPIMDMIDWGRSHPDNIRQCCSVYGIDAGRSIFVANLESAVSDTGKEILREHLLLVADSLSVTGEFVALNAKGWSKQRQVESTPAPFTQACFSSPSQCFLKAAKEGVRDDLQGSIDALAWGKVPGFGTGDQFEIIISPKVHGFTTPVDVYDLLSSTKTMRRTNSAPKSDKATVQPFGLLHSAFLKDIKVLDGKGIPMSLLRTIFTWKNIELLSQSLKRILHSYEINELLNERDEGLVKMVLQLHPNSVEKIGPGVKGIRVAKSKHGDSCCFEVVRIDGTFEDFSYHKCVLGATKIIAPKKMNFYKSKYLKNGTLESGGFSENP

Length 1453aa

Unique 552/1453=38%

Total=38%

V.PVGTLTSIGFSISNNNDR.D

K.MSVLEVEAPNQVTDSR.L

R.LGLPNPDSVCR.T

K.EVAALLNK.I

K.EVAALLNK.I

K.KQFQITEDQPER.C

K.QFQITEDQPER.C

R.YCTLNTGYPLMK.F

R.SGIVVEVNEESLMK.L

R.VTEIVHQFNGAR.L

K.LVGFEGNTLELSSR.V

R.VMECMQYSR.L

R.VMECMQYSR.L

R.LFSETVSSSK.D

R.LFSETVSSSK.D

R.TVVVGDPSLK.L

R.TVVVGDPSLK.L

K.LNEIGIPESIAK.R

K.LNEIGIPESIAK.R

R.LQVSEHLNQCNK.E

R.LVTSFVPTLLDNK.E

R.LVAIQVNDLQTGDK.I

R.LVAIQVNDLQTGDK.I

R.GDFDGDCLHGYVPQSIQAK.V

R.GDFDGDCLHGYVPQSIQAK.V

K.VELDELVALDK.Q

K.VELDELVALDK.Q

K.NCYLNR.A

K.NCYLNR.A

R.DGEGNFIER.L

R.DGEGNFIER.L

R.KNLTEEISYGLR.E

K.NLTEEISYGLR.E

K.NLTEEISYGLR.E

K.QQLMVESWR.D

K.QQLMVESWR.D

R.DFLAVNGEDK.E

R.DFLAVNGEDKEEDSVSDLAR.F

R.DFLAVNGEDKEEDSVSDLAR.F

K.SATLSELAVSAFK.D

K.SATLSELAVSAFK.D

K.DAYRDVQALAYR.Y

R.DVQALAYR.Y

R.DVQALAYR.Y

R.YGDQSNSFLIMSK.A

R.YGDQSNSFLIMSK.A

R.ELTCAAWNDPNSPLR.G

R.DSSFSGNADLPGTLSR.R

R.DIYAAYDGTVR.N

R.DIYAAYDGTVR.N

K.NVLECGSK.K

K.NVLECGSKK.G

R.EQTMSLYLSEYLSK.K

K.HGFEYGSLEIK.N

K.HGFEYGSLEIK.N

K.QLSAESVVSSLNEQYK.S

K.QLSAESVVSSLNEQYK.S

K.HSVLELDAIR.L

K.HSVLELDAIR.L

R.NGNHLAGELYLK.V

K.VTMYGDR.G

K.VTMYGDR.G

R.QCCSVYGIDAGR.S

R.SIFVANLESAVSDTGK.E

R.QVESTPAPFTQACFSSPSQCFLK.A

R.QVESTPAPFTQACFSSPSQCFLK.A

R.DDLQGSIDALAWGK.V

K.NIELLSQSLK.R

K.NIELLSQSLK.R

K.MVLQLHPNSVEK.I

K.HGDSCCFEVVR.I

R.IDGTFEDFSYHK.C

**NRPD2/E2 (AT3G23780)**

MPDMDIDVKDLEEFEATTGEINLSELGEGFLQSFCKKAATSFFDKYGLISHQLNSYNYFIEHGLQNVFQSFGEMLVEPSFDVVKKKDNDWRYATVKFGEVTVEKPTFFSDDKELEFLPWHARLQNMTYSARIKVNVQVEVFKNTVVKSDKFKTGQDNYVEKKILDVKKQDILIGSIPVMVKSILCKTSEKGKENCKKGDCAFDQGGYFVIKGAEKVFIAQEQMCTKRLWISNSPWTVSFRSENKRNRFIVRLSENEKAEDYKRREKVLTVYFLSTEIPVWLLFFALGVSSDKEAMDLIAFDGDDASITNSLIASIHVADAVCEAFRCGNNALTYVEQQIKSTKFPPAESVDECLHLYLFPGLQSLKKKARFLGYMVKCLLNSYAGKRKCENRDSFRNKRIELAGELLEREIRVHLAHARRKMTRAMQKHLSGDGDLKPIEHYLDASVITNGLSRAFSTGAWSHPFRKMERVSGVVANLGRANPLQTLIDLRRTRQQVLYTGKVGDARYPHPSHWGRVCFLSTPDGENCGLVKNMSLLGLVSTQSLESVVEKLFACGMEELMDDTCTPLFGKHKVLLNGDWVGLCADSESFVAELKSRRRQSELPREMEIKRDKDDNEVRIFTDAGRLLRPLLVVENLQKLKQEKPSQYPFDHLLDHGILELIGIEEEEDCNTAWGIKQLLKEPKIYTHCELDLSFLLGVSCAVVPFANHDHGRRVLYQSQKHCQQAIGFSSTNPNIRCDTLSQQLFYPQKPLFKTLASECLKKEVLFNGQNAIVAVNVHLGYNQEDSIVMNKASLERGMFRSEQIRSYKAEVDAKDSEKRKKMDELVQFGKTHSKIGKVDSLEDDGFPFIGANMSTGDIVIGRCTESGADHSIKLKHTERGIVQKVVLSSNDEGKNFAAVSLRQVRSPCLGDKFSSMHGQKGVLGYLEEQQNFPFTIQGIVPDIVINPHAFPSRQTPGQLLEAALSKGIACPIQKEGSSAAYTKLTRHATPFSTPGVTEITEQLHRAGFSRWGNERVYNGRSGEMMRSMIFMGPTFYQRLVHMSEDKVKFRNTGPVHPLTRQPVADRKRFGGIKFGEMERDCLIAHGASANLHERLFTLSDSSQMHICRKCKTYANVIERTPSSGRKIRGPYCRVCVSSDHVVRVYVPYGAKLLCQELFSMGITLNFDTKLC

Length 1172aa

Unique 287/1172=24.5%

Total=24.5%

| M.PDMDIDVK.D |  |
| --- | --- |
| M.PDMDIDVK.D |  |
| K.KAATSFFDK.Y |  |
| K.AATSFFDK.Y |  |
| K.AATSFFDK.Y |  |
| K.FGEVTVEKPTFFSDDK.E | |
| R.LQNMTYSAR.I |  |
| R.LQNMTYSAR.I |  |
| K.VNVQVEVFK.N |  |
| K.VNVQVEVFK.N |  |
| K.KGDCAFDQGGYFVIK.G | |
| K.GDCAFDQGGYFVIK.G | |
| K.VFIAQEQMCTK.R |  |
| K.VFIAQEQMCTK.R |  |
| R.CGNNALTYVEQQIK.S | |
| R.FLGYMVK.C |  |
| K.CLLNSYAGK.R |  |
| K.CLLNSYAGK.R |  |
| K.RIELAGELLER.E |  |
| R.IELAGELLER.E |  |
| R.IELAGELLER.E |  |
| R.VSGVVANLGR.A |  |
| R.VSGVVANLGR.A |  |
| R.ANPLQTLIDLR.R |  |
| R.QQVLYTGK.V |  |
| R.VCFLSTPDGENCGLVK.N | |
| R.PLLVVENLQK.L |  |
| R.VLYQSQK.H |  |
| R.VLYQSQK.H |  |
| K.TLASECLK.K |  |
| K.TLASECLK.K |  |
| K.TLASECLKK.E |  |
| K.KMDELVQFGK.T |  |
| K.MDELVQFGK.T |  |
| K.MDELVQFGK.T |  |
| K.VVLSSNDEGK.N |  |
| K.VVLSSNDEGK.N |  |
| K.VVLSSNDEGKNFAAVSLR.Q | |
| K.NFAAVSLR.Q |  |
| K.NFAAVSLR.Q |  |
| R.QTPGQLLEAALSK.G | |
| K.GIACPIQK.E |  |
| K.GIACPIQK.E |  |
| R.LVHMSEDK.V |  |
| R.LVHMSEDK.V |  |
| R.DCLIAHGASANLHER.L | |
| R.DCLIAHGASANLHER.L | |
| K.TYANVIER.T |  |
| K.TYANVIER.T |  |
| R.VCVSSDHVVR.V |  |
| R.VYVPYGAK.L |  |

Underlined peptides also map to the predicted protein product of the NRPD2b pseudogene (At3g18090), but to no other proteins and are thus included in the unique % coverage calculation as was done in [1].

**NRPB3/D3/E3A (At2g15430)**

MDGATYQRFPKIK~~IRELKDDYAK~~FELR~~ETDVSMANALR~~RVMISEVPTVAIDLVEIEVNSSVLNDEFIAHRLGLIPLTSERAMSMRFSRDCDACDGDGQCEFCSVEFRLSSK~~CVTDQTLDVTSR~~DLYSADPTVTPVDFTIDSSVSDSSEHKGIIIVKLRRGQELKLRAIARKGIGKDHAKWSPAATVTFMYEPDIIINEDMMDTLSDEEK~~IDLIESSPTK~~VFGMDPVTRQVVVVDPEAYTYDEEVIKKAEAMGKPGLIEISPKDDSFIFTVESTGAVKASQLVLNAIDLLKQKLDAVRLSDDTVEADDQFGELGAHMRGG

Length 319aa

Unique 141/319= 44.2% ;The remaining 13.5% also map to NRPD3B/E3B (AT2G15400).

Total 141+43=184/319=57.7%

| K.~~IRELKDDYAK~~.F |
| --- |
| R.~~ELKDDYAK~~.F |
| R.~~ETDVSMANALR~~.R |
| R.~~ETDVSMANALR~~.R |
| R.LGLIPLTSER.A |
| R.LGLIPLTSER.A |
| R.DCDACDGDGQCEFCSVEFR.L |
| R.DCDACDGDGQCEFCSVEFR.L |
| K.~~CVTDQTLDVTSR~~.D |
| R.DLYSADPTVTPVDFTIDSSVSDSSEHK.G |
| K.~~IDLIESSPTK~~.V |
| K.~~IDLIESSPTK~~.V |
| K.VFGMDPVTR.Q |
| K.VFGMDPVTR.Q |
| R.QVVVVDPEAYTYDEEVIK.K |
| R.QVVVVDPEAYTYDEEVIK.K |
| R.QVVVVDPEAYTYDEEVIKK.A |
| R.QVVVVDPEAYTYDEEVIKK.A |
| K.AEAMGKPGLIEISPK.D |
| K.PGLIEISPK.D |
| K.DDSFIFTVESTGAVK.A |
| K.QKLDAVR.L |
| R.LSDDTVEADDQFGELGAHMR.G |

**NRPD3B/E3B (At2g15400)**

MDGVTYQRFPTVK~~IRELKDDYAK~~FELR~~ETDVSMANALR~~RVMISEVPTMAIHLVKIEVNSSVLNDEFIAQRLSLIPLTSERAMSMRFCQDCEDCNGDEHCEFCSVEFPLSAK~~CVTDQTLDVTSR~~DLYSADPTVTPVDFTSNSSTSDSSEHKGIIIAKLRRGQELKLKALARKGIGKDHAKWSPAATVTYMYEPDIIINEEMMNTLTDEEK~~IDLIESSPTK~~VFGIDPVTGQVVVVDPEAYTYDEEVIKKAEAMGKPGLIEIHPKHDSFVFTVESTGALKASQLVLNAIDILKQKLDAIRLSDNTVEADDQFGELGAHMREG

Length 319aa

Unique 79/319=24.76% ; The remaining 13.5% also map to NRPB3/D3/E3A (At2g15430).

Total 79+43=122/319=38.24%

| K.~~IRELKDDYAK~~.F | |  |
| --- | --- | --- |
| R.~~ELKDDYAK~~.F | |  |
| R.~~ETDVSMANALR~~.R | |  |
| R.~~ETDVSMANALR~~.R | |  |
| K.IEVNSSVLNDEFIAQR.L | |  |
| K.IEVNSSVLNDEFIAQR.L | |  |
| R.LSLIPLTSER.A | |  |
| R.FCQDCEDCNGDEHCEFCSVEFPLSAK.C | |  |
|  | K.~~CVTDQTLDVTSR~~.D | |
| * | R.DLYSADPTVTPVDFTSNSSTSDSSEHK.G | |
| * | R.DLYSADPTVTPVDFTSNSSTSDSSEHK.G | |
|  | K.~~IDLIESSPTK~~.V | |
|  | K.~~IDLIESSPTK~~.V | |

**NRPD4/E4 (At4g15950)**

MSEKGGKGLKSSLKSKDGGKDGSSTKLKKGRKIHFDQRTPPANYKILNVSSDQQPFQSSAAKCGKSDKPTKSSKNSLHSFELKDLPENAECMMDCEAFQILDGIKGQLVGLSEDPSIKIPVSYDRALAYVESCVHYTNPQSVRKVLEPLKTYGISDGEMCVIANASSESVDEVLAFIPSLKTKKEVINQPLQDALEELSKLKKSE

Length 205 aa

Unique 26/205=12.7%

Total=12.7%

| K.ILNVSSDQQPFQSSAAK.C |
| --- |
| K.NSLHSFELK.D |
| K.NSLHSFELK.D |

**NRPB5/D5 (At3g22320)**

MLTEEELKRLYRIQKTLMQMLRDRGYFIADSELTMTKQQFIRKHGDNMKREDLVTLKAKRNDNSDQLYIFFPDEAKVGVKTMKMYTNRMKSENVFRAILVVQQNLTPFARTCISEISSKFHLEVFQEAEMLVNIKEHVLVPEHQVLTTEEKKTLLERYTVKETQLPRIQVTDPIARYFGLKRGQVVKIIRPSETAGRYVTYRYVV

Length 205aa

Unique 92/205=44.9%

Total=44.9%

| -.MLTEEELK.R |
| --- |
| -.MLTEEELK.R |
| -.MLTEEELKR.L |
| R.DRGYFIADSELTMTK.Q |
| R.GYFIADSELTMTK.Q |
| K.REDLVTLK.A |
| R.EDLVTLK.A |
| R.NDNSDQLYIFFPDEAK.V |
| R.TCISEISSK.F |
| R.TCISEISSK.F |
| K.EHVLVPEHQVLTTEEK.K |
| R.IQVTDPIAR.Y |
| R.IQVTDPIAR.Y |
| K.IIRPSETAGR.Y |

**NRPD5B/E5B (At2g41340)**

MEGKGKEIVVGHSISKSSVECHKYYLARRTTMEMLRDRGYDVSDEDINLSLQQFRALYGEHPDVDLLRISAKHRFDSSKKISVVFCGTGIVKVNAMRVIAADVLSRENITGLILVLQSHITNQALKAVELFSFKVELFEITDLLVNVSKHVLRPKHQVLNDKEKESLLKKFSIEEKQLPRLSSKDPIVRYYGLETGQVMKVTYKDELSESHVTYRCVS

Length 218aa

Unique 78/218=35.8%

Total=35.8%

| R.DRGYDVSDEDINLSLQQFR.A |
| --- |
| R.GYDVSDEDINLSLQQFR.A |
| R.ALYGEHPDVDLLR.I |
| R.VIAADVLSR.E |
| K.AVELFSFK.V |
| K.HQVLNDKEK.E |
| R.LSSKDPIVR.Y |
| R.YYGLETGQVMK.V |

**NRPB6A/D6A/E6A (At5g51940)**

MADEDYNDVDDLGYEDEPAEPEIEEGVEEDVEMKENDDVNGEPIEAEDKVETEPVQRPRKTSKFMTKYERARILGTRALQISMNAPVMVELEGETDPLEIAMKELRQRKIPFTIRRYLPDGSFEEWGVDELIVEDSWKRQVGGD

Length 144aa

Unique 25/144=17.4%

Total=17.4%

| K.ENDDVNGEPIEAEDKVETEPVQRPR.K |
| --- |
| K.VETEPVQRPR.K |

The VETEPVQRPR peptide also maps to NRPB6B/E6B (AT2G04630), but no peptides unique to this protein were identified.

**NRPD7A (At3g22900)**

MFIKVKLPWDVTIPAEDMDTGLMLQRAIVIRLLEAFSKEKATKDLGYLITPTILENIGEGKIKEQTGEIQFPVVFNGICFKMFKGEIVHGVVHKVHKTGVFLKSGPYEIIYLSHMKMPGYEFIPGENPFFMNQYMSRIQIGARVRFVVLDTEWREAEKDFMALASIDGDNLGPF

Length 174aa

Unique 34/174=19.5%

Total=19.5%

| R.LLEAFSK.E |
| --- |
| K.DLGYLITPTILENIGEGK.I |
| R.FVVLDTEWR.E |

**NRPD7B/E7B (At4g14660)**

MFLKVQLPWNVMIPAENMDAKGLMLKRAILVELLEAFASKKATKELGYYVAVTTLDKIGEGKIREHTGEVLFPVMFSGMTFKIFKGEIIHGVVHKVLKHGVFMRCGPIENVYLSYTKMPDYKYIPGENPIFMNEKTSRIQVETTVRVVVIGIKWMEVEREFQALASLEGDYLGPLSEE

Length 178aa

Unique 26/178=14.6%

Total=14.6%

| R.CGPIENVYLSYTK.M |
| --- |
| K.YIPGENPIFMNEK.T |

**NRPB9A/D9A/E9A (At3g16980)**

MSTMKFCR~~ECNNILYPKEDK~~EQK~~ILLYACR~~NCDHQEVADNSCVYRNEVHHSVSERTQILTDVASDPTLPRTKAVRCSKCQHREAVFFQATARGEEGMTLFFVCCNPNCGHRWRE

Length 114aa

Unique 50/114=43.9% ; The remaining 16.7% also map to NRPB9B/D9B/E9B (AT4G16265)

Total 50+19=69/114=60.5%

|  | R.~~ECNNILYPK~~.E | |
| --- | --- | --- |
|  | R.~~ECNNILYPK~~.E | |
|  | R.~~ECNNILYPKEDK~~.E | |
|  | K.~~ILLYACR~~.N | |
| R.NCDHQEVADNSCVYR.N | |  |
| R.NEVHHSVSER.T | |  |
| R.TQILTDVASDPTLPR.T | |  |
| R.EAVFFQATAR.G | |  |

**NRPB9B/D9B/E9B (At4g16265)**

MSTMKFCR~~ECNNILYPKEDK~~EQS~~ILLYACR~~NCDHQEAADNNCVYRNEVHHSVSEQTQILSDVASDPTLPRTKAVRCAKCQHGEAVFFQATARGEEGMTLFFVCCNPNCSHRWRE

Length 114aa

Unique 53/114=46.5%; The remaining 7.9% also map to NRPB9A/D9A/E9A (At3g16980).

Total 53+9=62/114=54.4%

|  | R.~~ECNNILYPK~~.E | |
| --- | --- | --- |
|  | R.~~ECNNILYPK~~.E | |
| R.~~ECNNILYPKEDK~~.E | |  |
| K.EDKEQSILLYACR.N | |  |
| S.~~ILLYACR~~.N | |  |
| R.NCDHQEAADNNCVYR.N | |  |
| R.NEVHHSVSEQTQILSDVASDPTLPR.T | |  |

**NRPB10/D10/E10 (At1g11475)**

MIIPVRCFTCGKVIGNKWDQYLDLLQLDYTEGDALDALQLVRYCCRRMLMTHVDLIEKLLNYNTLEKSDNS

Length 71aa

Unique 9/71=12.7%

Total=12.7%

| K.LLNYNTLEK.S |
| --- |
| K.LLNYNTLEK.S |

**NRPB11/D11/E11 (At3g52090)**

MNAPERYERFVVPEGTKKVSYDRDTKIINAASFTVEREDHTIGNIVRMQLHRDENVLFAGYQLPHPLKYKIIVRDPKDWFRNLKVVYGCLETVNISWFKLLVLTIHTTSQSSPMQAYNQAINDLDKELDYLKNQFEAEVAKFSNQF

Length 146aa

Unique 38/146=26%

Total=26%

| R.FVVPEGTK.K |
| --- |
| K.IINAASFTVER.E |
| K.IINAASFTVER.E |
| R.EDHTIGNIVR.M |
| K.NQFEAEVAK.F |
| K.NQFEAEVAK.F |

**NRPB12/D12/E12 (At5g41010)**

MDPAPEPVTYVCGDCGQENTLKSGDVIQCRECGYRILYKKRTRRVVQYEAR

Length 51aa

Unique 38/51=74.5%

Total=74.5%

| -.MDPAPEPVTYVCGDCGQENTLK.S |
| --- |
| D.PAPEPVTYVCGDCGQENTLK.S |
| K.SGDVIQCR.E |
| K.SGDVIQCR.E |
| R.RVVQYEAR.- |
| R.VVQYEAR.- |
| R.VVQYEAR.- |

**RDR2 (At4g11130)**

MVSETTTNRSTVKISNVPQTIVADELLRFLELHLGEDTVFALEIPTTRDNWKPRDFARVQFTTLEVKSRAQLLSSQSKLLFKTHNLRLSEAYDDIIPRPVDPRKRLDDIVLTVGFPESDEKRFCALEKWDGVRCWILTEKRRVEFWVWESGDCYKIEVRFEDIIETLSCCVNGDASEIDAFLLKLKYGPKVFKRVTVHIATKFKSDRYRFCKEDFDFMWIRTTDFSGSKSIGTSTCFCLEVHNGSTMLDIFSGLPYYREDTLSLTYVDGKTFASAAQIVPLLNAAILGLEFPYEILFQLNALVHAQKISLFAASDMELIKILRGMSLETALVILKKLHQQSSICYDPVFFVKTQMQSVVKKMKHSPASAYKRLTEQNIMSCQRAYVTPSKIYLLGPELETANYVVKNFAEHVSDFMRVTFVEEDWSKLPANALSVNSKEGYFVKPSRTNIYNRVLSILGEGITVGPKRFEFLAFSASQLRGNSVWMFASNEKVKAEDIREWMGCFRKIRSISKCAARMGQLFSASRQTLIVRAQDVEQIPDIEVTTDGADYCFSDGIGKISLAFAKQVAQKCGLSHVPSAFQIRYGGYKGVIAVDRSSFRKLSLRDSMLKFDSNNRMLNVTRWTESMPCFLNREIICLLSTLGIEDAMFEAMQAVHLSMLGNMLEDRDAALNVLQKLSGENSKNLLVKMLLQGYAPSSEPYLSMMLRVHHESQLSELKSRCRILVPKGRILIGCMDEMGILEYGQVYVRVTLTKAELKSRDQSYFRKIDEETSVVIGKVVVTKNPCLHPGDIRVLDAIYEVHFEEKGYLDCIIFPQKGERPHPNECSGGDLDGDQFFVSWDEKIIPSEMDPPMDYAGSRPRLMDHDVTLEEIHKFFVDYMISDTLGVISTAHLVHADRDPEKARSQKCLELANLHSRAVDFAKTGAPAEMPYALKPREFPDFLERFEKPTYISESVFGKLYRAVKSSLAQRKPEAESEDTVAYDVTLEEAGFESFIETAKAHRDMYGEKLTSLMIYYGAANEEEILTGILKTKEMYLARDNRRYGDMKDRITLSVKDLHKEAMGWFEKSCEDEQQKKKLASAWYYVTYNPNHRDEKLTFLSFPWIVGDVLLDIKAENAQRQSVEEKTSGLVSI

Length 1133aa

Unique 337/1133=29.7%

Total=29.7%

| K.ISNVPQTIVADELLR.F |
| --- |
| R.VQFTTLEVK.S |
| R.VQFTTLEVK.S |
| R.AQLLSSQSK.L |
| R.AQLLSSQSK.L |
| R.LSEAYDDIIPR.P |
| R.LSEAYDDIIPRPVDPR.K |
| R.LDDIVLTVGFPESDEK.R |
| R.CWILTEK.R |
| K.EDFDFMWIR.T |
| R.EDTLSLTYVDGK.T |
| R.LTEQNIMSCQR.A |
| K.IYLLGPELETANYVVK.N |
| R.VTFVEEDWSK.L |
| R.VTFVEEDWSK.L |
| K.LPANALSVNSK.E |
| K.LPANALSVNSK.E |
| K.EGYFVKPSR.T |
| R.VLSILGEGITVGPK.R |
| R.GNSVWMFASNEK.V |
| R.EWMGCFR.K |
| R.MGQLFSASR.Q |
| R.MGQLFSASR.Q |
| R.AQDVEQIPDIEVTTDGADYCFSDGIGK.I |
| R.AQDVEQIPDIEVTTDGADYCFSDGIGK.I |
| R.DAALNVLQK.L |
| R.DAALNVLQK.L |
| R.KIDEETSVVIGK.V |
| K.IDEETSVVIGK.V |
| K.NPCLHPGDIR.V |
| K.GERPHPNECSGGDLDGDQFFVSWDEK.I |
| K.IIPSEMDPPMDYAGSRPR.L |
| R.LMDHDVTLEEIHK.F |
| R.EFPDFLER.F |
| R.EFPDFLER.F |
| R.FEKPTYISESVFGK.L |
| K.EAMGWFEK.S |
| K.EAMGWFEK.S |

**RDM4 (At2g30280)**

MDGVGESSTQNEVEEKPVIVRVKRKVGQSLLDAFWLEINERPLKRPTLDFSKLSISDSGERGPSVAEDVKPKKVLVRHLETVTDSETTADIIHSFFESDHNEKSCSKGKFEERKIAFKKDNRKEQRLTKSVQKQQIASENARFEQIWRSRKGNKEGIHEKCHFFDVIRVDTEERRDNAQEFTSLEDQKMLASFLPLLRECIPTAAEEIEADIQSSHTEEYVYDYYAVNEEMDISEDSSKNQFPLVIVEDEEEFCDGSDESDYDSEDSNAEDHPKTDYPEEEEEEEEEDDDDDDDDESEEEKSEASDESDDEETSKRHVRSVLGDDEFDDYAEDVYGYSESDEEFES

Length 346 aa

Unique 40/346=11.6%

Total=11.6%

| K.LSISDSGER.G |
| --- |
| K.LSISDSGER.G |
| R.GPSVAEDVKPK.K |
| R.FEQIWR.S |
| R.RDNAQEFTSLEDQK.M |
| R.DNAQEFTSLEDQK.M |
| R.DNAQEFTSLEDQK.M |

**CLASSY1 (At3g42670)**

MKRKHYFEFNHPFNPCPFEVFCWGTWKAVEYLRIENGTMTMRLLENGQVLDDIKPFQRLRIRSRKATLIDCTSFLRPGIDVCVLYQRDEETPEPVWVDARVLSIERKPHESECLCTFHVSVYIDQGCIGLEKHRMNKVPVLVGLNEIAILQKFCKEQSLDRYYRWRYSEDCSSLVKTRLNLGKFLPDLTWLLVTSVLKNIVFQIRTVHEKMVYQIVTDEDCEGSSSSLSAMNITVEDGVVMSKVVLFNPAEDTCQDSDVKEEIEEEVMELRRSKRRSGRPERYGDSEIQPDSKDGWVRMMPYRYNIWNVSSDDDDEEEDCEDDKDTDDDLYLPLSHLLRKKGSKKGFSKDKQREIVLVDKTERKKRKKTEGFSRSCELSVIPFTPVFEPIPLEQFGLNANSLCGGVSGNLMDEIDKYRSKAAKYGKKKKKKIEMEEMESDLGWNGPIGNVVHKRNGPHSRIRSVSRETGVSEEPQIYKKRTLSAGAYNKLIDSYMSRIDSTIAAKDKATNVVEQWQGLKNPASFSIEAEERLSEEEEDDGETSENEILWREMELCLASSYILDDHEVRVDNEAFHKATCDCEHDYELNEEIGMCCRLCGHVGTEIKHVSAPFARHKKWTTETKQINEDDINTTIVNQDGVESHTFTIPVASSDMPSAEESDNVWSLIPQLKRKLHLHQKKAFEFLWKNLAGSVVPAMMDPSSDKIGGCVVSHTPGAGKTFLIIAFLASYLKIFPGKRPLVLAPKTTLYTWYKEFIKWEIPVPVHLLHGRRTYCMSKEKTIQFEGIPKPSQDVMHVLDCLDKIQKWHAQPSVLVMGYTSFLTLMREDSKFAHRKYMAKVLR~~ESPGLLVLDEGHNPR~~STKSRLRKALMKVDTDLRILLSGTLFQNNFCEYFNTLCLARPKFVHEVLVELDKKFQTNQAEQKAPHLLENRARKFFLDIIAKKIDTKVGDERLQGLNMLRNMTSGFIDNYEGSGSGSGDVLPGLQIYTLLMNSTDVQHKSLTKLQNIMSTYHGYPLELELLITLAAIHPWLVKTTTCCAKFFNPQELLEIEKLKHDAKKGSKVMFVLNLVFRVVKREKILIFCHNIAPIRLFLELFENVFRWKRGRELLTLTGDLELFERGRVIDKFEEPGGQSRVLLASITACAEGISLTAASR~~VIMLDSEWNPSK~~TKQAIARAFRPGQQKVVYVYQLLSR~~GTLEEDKYR~~RTTWKEWVSSMIFSEEFVEDPSQWQAEKIEDDVLREIVEEDKVKSFHMIMKNEKASTGG

Length 1256aa

Unique 101/1256=8%; The remaining 2.9% also map to CLASSY2 (AT5G20420).

Total 101+36=137/1256=10.9%

| R.DEETPEPVWVDAR.V |
| --- |
| R.YSEDCSSLVK.T |
| R.EIVLVDK.T |
| R.ETGVSEEPQIYK.K |
| K.LIDSYMSR.I |
| K.DKATNVVEQWQGLK.N |
| K.ATNVVEQWQGLK.N |
| R.VDNEAFHK.A |
| R.~~ESPGLLVLDEGHNPR~~.S |
| R.VIDKFEEPGGQSR.V |
| R.~~VIMLDSEWNPSK~~.T |
| R.~~GTLEEDKYR~~.R |
| K.IEDDVLR.E |
| R.EIVEEDKVK.S |

**CLASSY2 (AT5G20420)**

MKKRGFYNLKHPFDPCPFEFFCSGTWKPVEYMRIEDGMMTIRLLENGYVLEDIRPFQRLRLRSRKAALSDCICFLRPDIDVCVLYRIHEDDLEPVWVDARIVSIERKPHESECSCKINVRIYIDQGCIGSEKQRINRDSVVIGLNQISILQKFYKEQSTDQFYRWRFSEDCTSLMKTRLSLGKFLPDLSWLTVTSTLKSIVFQIRTVQTKMVYQIVTDEEGSSSTLSSMNITLEDGVSLSKVVKFNPADILDDSQDLEIKQETDYYQEEDEVVELRRSKRRNVRPDIYTGCDYEPDTIDGWVRMMPYQFGKCAVNVESDEDEDDNNEDGDTNDDLYIPLSRLFIKKKKTNSREAKPKSRKGEIVVIDKRRVHGFGRKERKSELSVIPFTPVFEPIPLEQFGLNANSFGGGGSFSRSQYFDETEKYRSKGMKYGKKMTEMEEMMEADLCWKGPNQVKSFQKRTSRSSRSVAPKTEDSDEPRVYKKVTLSAGAYNKLIDTYMNNIESTIAAKDEPTSVVDQWEELKKTNFAFKLHGDMEKNLSEDGEGETSENEMLWREMELCLASSYILDDNEVRVDNEAFEKARSGCEHDYRLEEEIGMCCRLCGHVGSEIKDVSAPFAEHKKWTIETKHIEEDDIKTKLSHKEAQTKDFSMISDSSEMLAAEESDNVWALIPKLKRKLHVHQRRAFEFLWRNVAGSVEPSLMDPTSGNIGGCVISHSPGAGKTFLIIAFLTSYLKLFPGKRPLVLAPKTTLYTWYKEFIKWEIPVPVHLIHGRRTYCTFKQNKTVQFNGVPKPSRDVMHVLDCLEKIQKWHAHPSVLVMGYTSFTTLMREDSKFAHRKYMAKVLR~~ESPGLLVLDEGHNPR~~STKSRLRKALMKVGTDLRILLSGTLFQNNFCEYFNTLCLARPKFIHEVLMELDQKFKTNHGVNKAPHLLENRARKLFLDIIAKKIDASVGDERLQGLNMLKNMTNGFIDNYEGSGSGSGDALPGLQIYTLVMNSTDIQHKILTKLQDVIKTYFGYPLEVELQITLAAIHPWLVTSSNCCTKFFNPQELSEIGKLKHDAKKGSKVMFVLNLIFRVVKREKILIFCHNIAPIRMFTELFENIFRWQRGREILTLTGDLELFERGRVIDKFEEPGNPSRVLLASITACAEGISLTAASR~~VIMLDSEWNPSK~~TKQAIARAFRPGQQKVVYVYQLLSR~~GTLEEDKYR~~RTTWKEWVSCMIFSEEFVADPSLWQAEKIEDDILREIVGEDKVKSFHMIMKNEKASTG

Length 1261aa

Unique 149/1261=11.8% ; The remaining 2.85% also map to CLASSY1 (At3g42670).

Total 149+36=185/1261=14.7%

| * | R.IHEDDLEPVWVDAR.I |
| --- | --- |
| * | R.IYIDQGCIGSEK.Q |
| * | K.FNPADILDDSQDLEIK.Q |
| * | K.QETDYYQEEDEVVELR.R |
| * | K.VTLSAGAYNK.L |
| * | K.LIDTYMNNIESTIAAK.D |
| * | K.DEPTSVVDQWEELKK.T |
| * | K.NLSEDGEGETSENEMLWR.E |
|  | R.~~ESPGLLVLDEGHNPR~~.S |
| * | K.FFNPQELSEIGK.L |
| * | R.VIDKFEEPGNPSR.V |
|  | R.~~VIMLDSEWNPSK~~.T |
|  | R.~~GTLEEDKYR~~.R |
| * | K.IEDDILR.E |

**CHR31 (At1g05490)**

MECIGKRVKSRSWQRLQAVNKRKKMETVAPVTSPPKKRRQKKPKNYDSDIEDITPTCNDSVPPPQVSNMYSVPNNSVKESFSRIMRDLNVEKKSGPSSSRLTDGSEQNPCLKERSFRVSDLGVEKKCSPEITDLDVGIPVPRFSKLKDVSEQKNTCLMQKSSPEIADLDLVISVPSSSVLKDVSEEIRFLKDKCSPEIRGLVLEKSVPGEIEILSDSESETEARRRASAKKKLFEESSRIVESISDGEDSSSETDEEEEENQDSEDNNTKDNVTVESLSSEDPSSSSSSSSSSSSSSSSSSSDDESYVKEVVGDNRDDDDLRKASSPIKRVSLVERKALVRYKRSGSSLTKPRERDNKIQKLNHREEEKKERQREVVRVVTKQPSNVVYTCAHCGKENTGNPESHSSFIRPHSIRDEIEDVNNFASTNVSKYEDSVSINSGKTTGAPSRPEVENPETGKELNTPEKPSISRPEIFTTEKAIDVQVPEEPSRPEIYSSEKAKEVQAPEMPSRPEVFSSEKAKEIQVPEMPSIPEIQNSEKAKEVQANNRMGLTTPAVAEGLNKSVVTNEHIEDDSDSSISSGDGYESDPTLKDKEVKINNHSDWRILNGNNKEVDLFRLLVNSVWEKGQLGEEDEADELVSSAEDQSQEQAREDHRKYDDAGLLIIRPPPLIEKFGVEEPQSPPVVSEIDSEEDRLWEELAFFTKSNDIGGNELFSNVEKNISANETPAAQCKKGKHDLCIDLEVGLKCMHCGFVEREIRSMDVSEWGEKTTRERRKFDRFEEEEGSSFIGKLGFDAPNNSLNEGCVSSEGTVWDKIPGVKSQMYPHQQEGFEFIWKNLAGTIMLNELKDFENSDETGGCIMSHAPGTGKTRLTIIFLQAYLQCFPDCKPVIIAPASLLLTWAEEFKKWNISIPFHNLSSLDFTGKENSAALGLLMQKNATARSNNEIRMVKIYSWIKSKSILGISYNLYEKLAGVKDEDKKTKMVREVKPDKELDDIREILMGRPGLLVLDEAHTPRNQRSCIWKTLSKVETQKRILLSGTPFQNNFLELCNVLGLARPKYLERLTSTLKKSGMTVTKRGKKNLGNEINNRGIEELKAVMLPFVHVHKGSILQSSLPGLRECVVVLNPPELQRRVLESIEVTHNRKTKNVFETEHKLSLVSVHPSLVSRCKISEKERLSIDEALLAQLKKVRLDPNQSVKTRFLMEFVELCEVIKEKVLVFSQYIDPLKLIMKHLVSRFKWNPGEEVLYMHGKLEQKQRQTLINEFNDPKSKAKVFLASTK~~ACSEGISLVGASR~~VILLDVVWNPAVERQAISRAYRIGQKRIVYTYHLVAKGTPEGPKYCKQAQKDRISELVFACSSRHDKGKEKIAEAVTEDKVLDTMVEHSKLGDMFDNLIVQPKEADLVEGFSILMP

Length 1410aa

Unique 239/1410=16.9; The remaining 1% also map to CHR40 (At3g24340)

Total 239+13=252/1410=17.9%

| K.CSPEITDLDVGIPVPR.F |
| --- |
| K.EVQAPEMPSRPEVFSSEK.A |
| K.EIQVPEMPSIPEIQNSEK.A |
| K.GQLGEEDEADELVSSAEDQSQEQAR.E |
| K.GQLGEEDEADELVSSAEDQSQEQAR.E |
| K.SNDIGGNELFSNVEK.N |
| K.NISANETPAAQCK.K |
| R.SMDVSEWGEK.T |
| R.SMDVSEWGEK.T |
| R.FEEEEGSSFIGK.L |
| K.LGFDAPNNSLNEGCVSSEGTVWDK.I |
| K.NLAGTIMLNELK.D |
| K.NLGNEINNR.G |
| R.ECVVVLNPPELQR.R |
| R.QTLINEFNDPK.S |
| K.~~ACSEGISLVGASR~~.V |
| R.ISELVFACSSR.H |
| K.IAEAVTEDK.V |
| K.VLDTMVEHSK.L |
| K.LGDMFDNLIVQPK.E |

**CHR40 (At3g24340)**

MDMTSCVARRTRSRTESYLNSILNKSKGISGEEEDQSLGCVNSRTEKRRVNMRDACSPSPRKKKRRRRKDDDDDVVFVRTEYPEGKRDDENVGSTSGNLQSKSFDFGDRVCDFDADDRNLGCEEKASNFNPIDDDDDVVFVGTVQRENDHVEDDDNVGSASVISPRVCDFDEDDAKVSGKENPLSPDDDDDVVFLGTIAGENQHVEDVNAGSEVCDILLDDANLRGEEKTYVSDEVVSLSSSSDDEEDPLEELGTDSREEVSGEDRDSGESDMDEDANDSDSSDYVGESSDSSDVESSDSDFVCSEDEEGGTRDDATCEKNPSEKVYHHKKSRTFRRKHNFDVINLLAKSMLESKDVFKEDIFSWDKIAEVDSREDPVVRESSSEKVNEHGKPRERRSFHRVREKNHLNGESFYGGEKLCDGEETINYSTEDSPPLNLRFGCEEPVLIEKTEEEKELDSLWEDMNVALTLEGMHSSTPDKNGDMLCSKGTHDFVLDDEIGLKCVHCAYVAVEIKDISPAMDKYRPSVNDNKKCSDRKGDPLPNRLEFDASDPSSFVAPLDNIEGTVWQYVPGIKDTLYPHQQEGFEFIWKNLAGTTKINELNSVGVKGSGGCIISHKAGTGKTRLTVVFLQSYLKRFPNSHPMVIAPATLMRTWEDEVRKWNVNIPFYNMNSLQLSGYEDAEAVSRLEGNRHHNSIRMVKLVSWWKQKSILGISYPLYEKLAANKNTEGMQVFRRMLVELPGLLVLDEGHTPRNQSSLIWKVLTEVRTEKRIFLSGTLFQNNFKELSNVLCLARPADKDTISSRIHELSKCSQEGEHGRVNEENRIVDLKAMIAHFVHVHEGTILQESLPGLRDCVVVLNPPFQQKKILDRIDTSQNTFEFEHKLSAVSVHPSLYLCCNPTKKEDLVIGPATLGTLKRLRLKYEEGVKTKFLIDFIRISGTVKEKVLVYSQYIDTLKLIMEQLIAECDWTEGEQILLMHGKVEQRDRQHMIDNFNKPDSGSKVLLASTK~~ACSEGISLVGASR~~VVILDVVWNPSVESQAISRAFRIGQKRAVFIYHLMVKDTSEWNKYCKQSEKHRISELVFSSTNEKDKPINNEVVSKDRILDEMVRHEKLKHIFEKILYHPKKSDMNTSFF

Length 1132 aa

Unique 156/1132=13.8%; The remaining 1.1% also map to CHR31 (At1g05490).

Total 156+13=169/1132=14.9%

| K.GISGEEEDQSLGCVNSR.T |
| --- |
| K.DDDDDVVFVR.T |
| K.SFDFGDR.V |
| K.ASNFNPIDDDDDVVFVGTVQR.E |
| K.ASNFNPIDDDDDVVFVGTVQR.E |
| R.FGCEEPVLIEK.T |
| K.GTHDFVLDDEIGLK.C |
| K.DISPAMDK.Y |
| K.INELNSVGVK.G |
| K.NTEGMQVFR.R |
| K.EDLVIGPATLGTLK.R |
| K.VLVYSQYIDTLK.L |
| K.~~ACSEGISLVGASR~~.V |
| R.ISELVFSSTNEK.D |
| K.DKPINNEVVSK.D |

**SHH1 (At1g15215)**

MAASDDSSHYFTEFTLSEIVDMENLYKELGDQSLHKDFCQTVASTFSCSVNRNGKSSITWKQVQIWFQEKLKHQSQPKSKTLPSPPLQIHDLSNPSSYASNASNATFVGNSTFVQTRKGKASDLADLAFEAKSARDYAWYDVSSFLTYRVLRTGELEVRVRFSGFDNRHDEWVNVKTSVRERSIPVEPSECGRVNVGDLLLCFQEREDQALYCDGHVLNIKRGIHDHARCNCVFLVRYELDNTEESLGLERICRRPEE

Length 258aa

Unique 65/258=25.2%

Total=25.2%

| K.ELGDQSLHK.D |
| --- |
| K.ASDLADLAFEAK.S |
| R.FSGFDNR.H |
| R.HDEWVNVK.T |
| R.EDQALYCDGHVLNIK.R |
| R.YELDNTEESLGLER.I |

1. Ream TS, Haag JR, Wierzbicki AT, Nicora CD, Norbeck AD, et al. (2009) Subunit compositions of the RNA-silencing enzymes Pol IV and Pol V reveal their origins as specialized forms of RNA polymerase II. Mol Cell 33: 192-203.
