# Supplementary material for: SHH1, a Homeodomain Protein Required for DNA Methylation, As Well As RDR2, RDM4, and Chromatin Remodeling Factors, Associate with RNA Polymerase IV
Source: PLoS Genet. 2011 Jul 21;7(7):e1002195. doi: 10.1371/journal.pgen.1002195 (PMC3141008; doi:10.1371/journal.pgen.1002195)
Supplement: Table S1 — Amino acid sequences of epitope tags. The amino acid sequence of each tandem affinity epitope tag are shown with the Biotin Ligase Recognition Peptide (BLRP) in bold type, the L to R mutation in the mutated BLRP tag in red, the 3C protease cleavage site underlined and the Flag, Myc or HA tag in large, non-bold text. * indicates a stop codon. (DOC) [file pgen.1002195.s002.doc]

Table S1 **Amino acid sequences of epitope tags.** The amino acid sequence of each tandem affinity epitope tag are shown with the Biotin Ligase Recognition Peptide (BLRP) in bold type, the L to R mutation in the mutated BLRP tag in red, the 3C protease cleavage site underlined and the Flag, Myc or HA tag in large, non-bold text. * indicates a stop codon.

| **Epitope Tag** | **Amino Acid Sequence** |
| --- | --- |
| **Carboxy-terminal 3xFlag** | KLGTDYKDDDDKDYKDDDDKDYKDDDDKGSLEVLFQGPLEGS**MAGGLNDIFEAQRIEWHEDTGGS*** |
| **Carboxy-terminal 3xFlag-BLRP** | KLGTDYKDDDDKDYKDDDDKDYKDDDDKGSLEVLFQGPLEGS**MAGGLNDIFEAQKIEWHEDTGGS*** |
| **Carboxy-terminal 9xMyc** | GRAGTGRSRTSGEQKLISEEDLNGEQKLISEEDLNGEQKLISEEDLNGSSRGEQKLISEEDLNGEQKLISEEDLNGEQKLISEEDLNGSSRGEQKLISEEDLNGEQKLISEEDLNGEQKLISEEDLNGSTSGSLEVLFQGPLEGS**MAGGLNDIFEAQRIEWHEDTGGS*** |
| **Carboxy-terminal 3xMyc-BLRP** | GRAGTGRSRTSGEQKLISEEDLNGEQKLISEEDLNGEQKLISEEDLNGSTSGSLEVLFQGPLEGS**MAGGLNDIFEAQKIEWHEDTGGS*** |
| **Amino-terminal BLRP-3xHA** | RYH**MAGGLNDIFEAQKIEWHEDTGGS**SIPGLEVLFQGPLEMGYPYDVPDYAGMGYPYDVPDYAGMGYPYDVPDYAGMV |
