# Supplementary material for: SHH1, a Homeodomain Protein Required for DNA Methylation, As Well As RDR2, RDM4, and Chromatin Remodeling Factors, Associate with RNA Polymerase IV
Source: PLoS Genet. 2011 Jul 21;7(7):e1002195. doi: 10.1371/journal.pgen.1002195 (PMC3141008; doi:10.1371/journal.pgen.1002195)
Supplement: Table S2 — DNA sequences of primers and quantitative PCR probes. (DOC) [file pgen.1002195.s003.doc]

Table S2 **DNA sequences of primers and quantitative PCR probes.**

| **Locus** | **Primer numbers** | **Primer sequence 5’ to 3’** |  |
| --- | --- | --- | --- |
| ***NRPD1*** | 3912  3913 | CACCGGTGTCTCACATTCCAAAGTCCCC  CGGGTTTTCGGAGAAACCACC |  |
| ***RDR2*** | 7853  7854 | CACCAGCAGATAGTGAGTTCTTGG  AATGGATACAAGTCCACTTG |  |
| ***CLSY1*** | 7857  7859 | CACCGTACCTTTTCCATCCTTCTCTG  GCCACCTGTTGAAGCTTT |  |
| ***SHH1*** | 7927  7856 | CACCCAGAGAAAGAGATAAGCAAAGATGG  CTCTTCAGGGCGGCGGCA |  |
| ***SHH1 (At1g15215) (A)*** | 7971  7900 | CGCTCGATAATGGCTGCT  TCCCAACAAAGGTAGCATTTG |  |
| ***SHH1 (At1g15215) (B)*** | 7928  7856 | CGTGTACGGTTTTCTGGGTT CTCTTCAGGGCGGCGGCA |  |
| ***SHH1 (At1g15215) (C)*** | 7971  7856 | CGCTCGATAATGGCTGCT  CTCTTCAGGGCGGCGGCA |  |
| ***UBQ*** | 3483  3484 | GATCTTTGCCGGAAAACAATTGGAGG  CGACTTGTCATTAGAAAGAAAGAGAT |  |
| ***AtSN1*** | 6699  6700 | ACTTAATTAGCACTCAAATTAAACAAAATAAGT  TTTAAACATAAGAAGAAGTTCCTTTTTCATCTAC |  |
| ***FWA*** | 2004  2005 | GGTTTTATATTAATATTAAAGAGTTATGGGTYGAAGTTT  CAAAATACTTTACACATAAACRAAAAACAAACAAATCRAA |  |
| ***MEA-ISR*** | 3733  3734  M16 | CGCGAACGACTATTGCTAAA  TGAAATCTAACCGGATTTTGG  TGACCACGGTTAAATGGCGGG-HEX |  |
| ***IGN5*** | 6606  6607  M36 | TCCCGAGAAGAGTAGAACAAATGCTAAAA  CTGAGGTATTCCATAGCCCCTGATCC  TTGGGCCGAATAACAGCAAGTCC-HEX |  |
| ***ACTIN*** | 2452  2453  M17 | TCGTGGTGGTGAGTTTGTTAC  CAGCATCATCACAAGCATCC  TTTTCCCTAGTTGAGATGGGAATT-HEX |  |
